# Supplementary material for: Early-life social experience affects offspring DNA methylation and later life stress phenotype
Source: Nat Commun. 2021 Jul 20;12:4398. doi: 10.1038/s41467-021-24583-x (PMC8292380; doi:10.1038/s41467-021-24583-x)
Supplement: Supplementary file 1 — Supplementary Information [file 41467_2021_24583_MOESM1_ESM.pdf]

Supplementary Information

Early-life social experience affects offspring DNA methylation and later life stress phenotype.

Zachary M. Laubach[1,2,3,4*‡], Julia R. Greenberg[1,2,4], Julie W. Turner[1,2,3,4],
Tracy M. Montgomery[1,2,4], Malit O. Pioon[4], Maggie A. Sawdy[1,2], Laura Smale[1,5],
Raymond G. Cavalcante[6], Karthik R. Padmanabhan[6], Claudia Lalancette[6], Bridgett vonHoldt[7],
Christopher D. Faulk[8], Dana C. Dolinoy[6,9,10], Kay E. Holekamp[1,2,3,4], Wei Perng[11]

[*] Correspondence and requests for materials should be addressed to
zachary.laubach@colorado.edu

[‡]Current address: Department of Ecology and Evolutionary Biology, University of Colorado, Boulder, USA.

This PDF file includes:

Supplementary Figures 1-13
Supplementary Tables 1-8

Supplementary Methods

Maternal care behaviors from focal animal survey (FAS)

Our maternal care data included 1532 FAS totaling approximately 798 hours of observations of 258 mother-offspring pairs when offspring were ≤1 year old, which is the approximate age at weaning in this sample (11.59 months) as well as among spotted hyenas more generally [1]. We filtered these data to include only FAS sessions during which 1) the mother was lactating and 2) the mother and offspring were observed together for a minimum of five minutes. We focused on three specific maternal care behaviors: time spent in close proximity, nursing, and grooming. We chose these specific metrics of maternal care because comparable behaviors in primates and rodents have been shown to influence offspring behavior and physiology, albeit in captive settings. Additionally, these behaviors capture the effect of nutrition as well as physical contact on offspring development. *Nota bene*, maternal care behaviors were not mutually exclusive in their occurrence; for example, a portion of time spent in close proximity includes time spent nursing or grooming.

Given that we had anywhere from 1 to 29 repeated observations of mother-offspring interactions for each metric of interest, we converted the repeated measurements into a single value for use as explanatory variables in the regression models. To do this, we fit generalized linear mixed-effects models where the repeated outcome was counts of each maternal care behavior, and fixed effects (i.e., independent variables) included characteristics that are known to affect mother-offspring interactions – namely, the offspring's age in months on the date of the FAS, timing of the FAS (morning or evening), and FAS season (during the annual wildebeest and zebra migration, present or absent). We also included a random effect for offspring ID to account for correlations among the repeated observations, and an offset of the natural log of

the length of time the mother-offspring were both present during the FAS to control for observer effort (*Nota bene*: the offset also facilitates interpretation of parameter estimates such that they reflect incident rates or proportions of time spent engaged in a particular behavior in relation to the total time the mother-offspring pair was observed).

Because behavioral count data are over-dispersed and zero-inflated, we assessed model fit of the mixed-effects models using three different underlying distributions with vs. without a zero-inflation correction for a total of six model specifications per type of maternal care behavior. We used the R package glmmTMB to fit a Poisson distributed model in which the mean equals the variance, as well as two parameterizations of negative binomial distributed models which differ in how the variance scales with respect to the mean [2,3]. More specifically, in the negative binomial 1 models, the variance is scaled as a multiplicative function of the mean and an estimated dispersion parameter, while in the negative binomial 2 models the variance is scaled as a quadratic function of the mean and an estimated dispersion parameter [4]. We used a simple form of a zero-inflation correction in which each observation has an equal probability of being a zero [2]. We compared model fit using AIC for each distribution with and without modeling zero-inflation and selected the model with the lowest AIC (Supplementary Table 1). This model was then used to extract residuals – i.e., the Best Linear Unbiased Predictors (BLUPs), which represent the deviation of each maternal care behavior received by each hyena relative to the population average for that particular behavior. We added the individual random effects to the overall model intercept, which represents a relevant population-level biological anchor (i.e., the average amount of maternal care received by the overall population when all other variables are set to the referent level). After appending the BLUPs to the intercept, we exponentiated the variable to transform the estimates from the

natural log scale back to the original scale, proportion of minutes during which the mother-infant pair were observed together. Finally, we z-score standardized all maternal care BLUPs for comparability purposes prior to using these measures as explanatory variables in downstream models. We note that for the mERRBS part of the study we restricted the maternal care data to a time period that roughly overlapped with the mERRBS data collection and reextracted maternal care BLUPs.

Supplementary Table 1. AIC values for generalized linear mixed models and zero-inflated generalized linear mixed-models of maternal care behaviors. The best fitting model (lowest AIC) was used to generate Best Unbiased Linear Predictors (BLUPs) for each hyena offspring.

Maternal Care behaviors	dAIC	Deg. freedom
Close proximity		
Zero-inflated negative binomial (1) distributed model [†,‡]	0.0	7
Zero-inflated negative binomial (2) distributed model [†,§]	3.6	7
Negative binomial (1) distributed model	390.2	6
Negative binomial (2) distributed model	595.4	6
Zero-inflated Poisson distributed model [†]	1563.5	6
Poisson distributed model	4370.8	5
Nursing		
Zero-inflated negative binomial (1) distributed model [†,‡]	0.0	7
Zero-inflated negative binomial (2) distributed model [†,§]	17.1	7
Negative binomial (1) distributed model	754.4	6
Zero-inflated Poisson distributed model	791.7	6
Negative binomial (2) distributed model	1059.5	6
Poisson distributed model	9486.6	5
Grooming		
Zero-inflated negative binomial (1) distributed model [†,‡]	0.0	7
Negative binomial (1) distributed model	11.8	6
Zero-inflated negative binomial (2) distributed model [†,§]	32.7	7
Negative binomial (2) distributed model	38.9	6
Zero-inflated Poisson distributed model [†]	114.6	6
Poisson distributed model	736.7	5

Models are adjusted for offspring hyena's age (months), time of day, and migration season on the date of the FAS.

Models include a random intercept for offspring ID.

[†] Single zero inflation parameter applied to all observations.

[‡] Negative binomial 1 where, variance = $(1+\alpha)\mu$.

[§] Negative binomial 2 where, variance = $\mu + \alpha\mu^2$

Our FAS data were collected during four distinct study periods between 1988 and 2013. Therefore, we checked for consistency between the four periods of data collection by visually inspecting a principal component analysis (PCA) plot (Supplementary Figure 1). We saw no evidence of clustering by sample collection period and concluded that variation in maternal care was likely not confounded by sampling batch effects, so we were able to pool data from all study periods together.

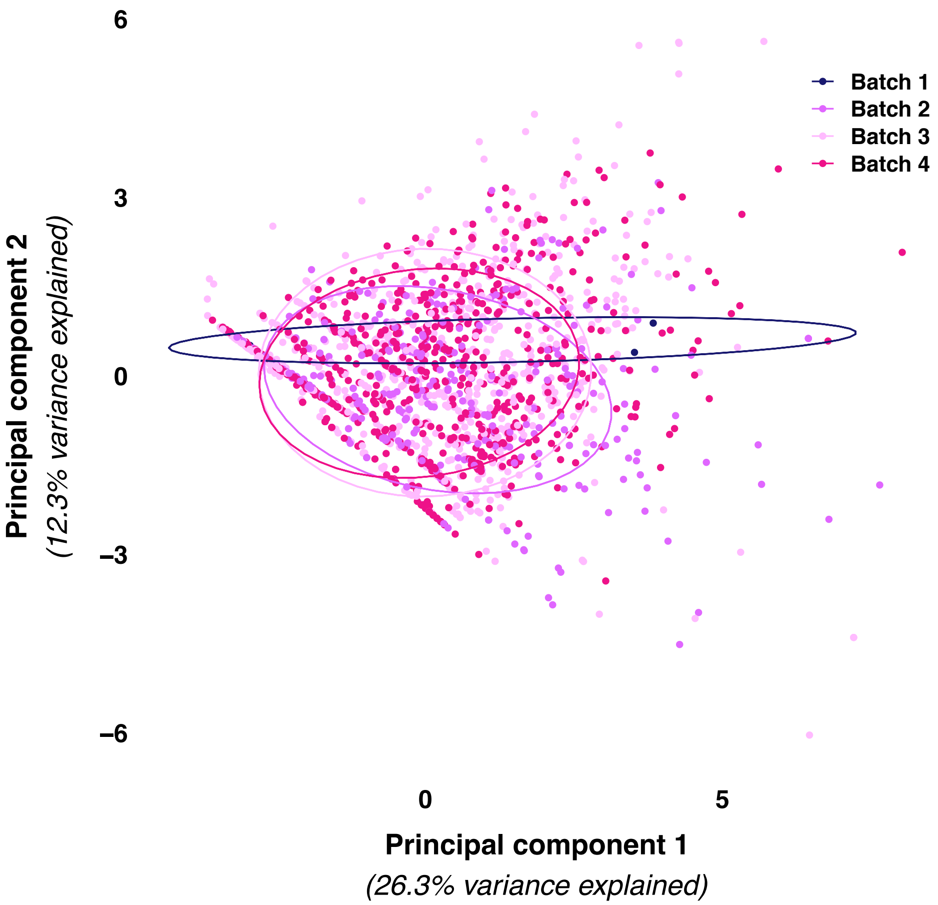

Supplementary Figure 1. Principal Components Analysis (PCA) plot of focal animal survey (FAS) batches (data collection groupings) for maternal care behaviors among N = 258 mother-offspring pairs. Ellipses represent 68% probability. Batches represent unique study periods. A square root transformation was used on count data prior to the PCA.

Social network metrics derived from 115 hyenas during the communal den dependent (CD) and communal den independent (DI) periods of development

Association social networks were based on regular 15-20 minute interval scan sampling during observations sessions in which two or more hyenas were present [5]. The networks incorporated twice weighted association index data [6], which enabled us to correct for sampling bias that could stem from variation in hyena group observability, thus providing more reliable information about social bonds in our population [5,7]. In our association networks, degree centrality (i.e., degree) corresponded to the number of different individuals with which the hyena was recorded in the same session. Strength, a weighted metric of degree or network connectedness [8], corresponded to the total number of times the hyena was observed with other clan mates, including repeated associations with the same individuals. Betweenness centrality (i.e., betweenness) was calculated as the number of shortest paths that connected hyenas in the group and passed through the hyena of interest [8]. An individual with high betweenness can be thought of as a bridge by which otherwise unconnected members of the network are indirectly connected. As with maternal care, we z-score standardized all association index social network metrics prior to using these measures as explanatory variables in downstream models.

Blood collection, processing, and storage

Hyenas from our study population were immobilized using a CO_2 rifle that propelled a pressurized dart containing 6.5 mg/kg of tiletamine-zolazepam (Telazol ®). After the hyena was sedated, we drew blood from their jugular vein into ethylenediaminetetraacetic acid (EDTA) coated vacuum tubes. We flash froze whole blood samples in liquid nitrogen or we extracted

genomic DNA using Gentra Pure Gene or PAXgene Blood DNA kits by Qiagen® and stored samples until they were transported to the U.S. for long term storage at -80°C.

Global (%CCGG) DNA methylation

We used the LUminometric Methylation Assay (LUMA) [9,10] to measure global DNA methylation. Our laboratory procedure, and data processing have been described previously [11]. In brief, this assay uses a parallel enzyme digestion of DNA to quantify the amount of methylated vs unmethylated CpG sites within the '5-CCGG-3' recognition sequence. Following pyrosequencing of the enzyme-digested DNA, we calculated a composite global methylation value for each hyena representing the average DNA methylation across the hyena genome.

We annotated 'CCGG' motifs in the draft hyena genome using the UCSC Genome Browser Kentutils tool, 'cpg_lh' to first identify CpG islands [12]. Then we used the Bioconductor package, 'annotatr' to map the 'CCGG' motifs to the hyena genome [13]. We categorized CpG shores as the 2kb regions flanking the start and end points on both sides of the CpG islands. CpG shelves were designated as the 2kb region flanking the up and downstream regions of the CpG shelves. All other parts of the genome were designated as the interCGI region. Similarly, we used the annotatr package in combination with the draft hyena genome general feature format (GFF) file [14] to classify regions of the genome as promoter, exon, intron and intergenic and summarized the frequency of 'CCGG' motifs in each of these regions.

Genome-wide DNA methylation: multiplexed Enhanced Reduced Representation Bisulfite Sequencing (mERRBS)

Genomic library preparation and sequencing

First, we digested approximately 100ng of genomic DNA for 16-18 hours with the restriction enzyme MSpI. Digested DNA was purified via phenol-chloroform extraction and ethanol precipitation. Next, we ran the digested DNA through an end-repair reaction, and an A-tailing reaction, which adenylated the 3' of the DNA. Third, we ligated paired-end methylated adapters to the A-tailed DNA and incubated at 16°C overnight [15]. Next, we selected the ligated DNA fragments in the ranges of 150-250 bp and 250-450 bp from an agarose gel and purified the excised fragments with a Qiagen QIAquick® Gel Extraction kit. Size-selected DNA was bisulfite treated using Zymo Easy DNA Methylation™ kits, followed by PCR enrichment of the bisulfite converted DNA using the Roche FastStart™ High Fidelity PCR system. Prior to sequencing, DNA libraries were cleaned using AMPure XPSPRI beads, and DNA library quantity and quality were assessed with a Qubit® High Sensitivity dsDNA kit and Agilent's High Sensitivity D1000 Tape screen, respectively. Finally, we multiplexed five libraries per flow cell on an Illumina HiSeq4000® platform for single-end sequencing with a 50-nucleotide read length. Included alongside of the hyena samples were libraries for a human genomic DNA sample and hyena samples were spiked with a lambda phage DNA sample to serve as a control and to estimate bisulfite conversion efficiency. More detailed information on the mERRBS library preparation and sequencing protocol, can be found in Garrett-Bakelman et al. (2015).

Bioinformatics pipeline

We assessed the quality of raw mERRBS data and identified specific reads from each sequenced sample that required trimming using FastQC (FastQC) (v0.11.3). We used TrimGAlore (Trim Galore!) (v0.4.5) to remove low quality bases with Phred quality scores < 20,

as well as adapter sequences, primers and extra bases from the 3' ends of reads that were the product of poly-A tail end-repair. Next, we used Bismark (v0.19.0) to perform alignment and methylation calling (identification of methylated vs unmethylated CpG sites) of sequenced short reads [17]. In this program, residual cytosines are converted to thymines in both the sequenced reads and the reference spotted hyena genome [14]. Using Bowtie2 (v2.3.4) [18] within Bismark, we then aligned short reads using the default parameters (multi-seed length of 20bp with 0 mismatches) and methylation calls were retained for all nucleotides with a read depth ≥ 5. Prior to downstream statistical analysis, we further restricted the data set to nucleotides with a read depth ≥ 10 and we used methylKit to exclude nucleotides that did not have coverage across all samples [19].

Candidate gene DNA methylation

We used a target gene approach to measure DNA methylation of CpGs within the putative promoter region of the spotted hyena glucocorticoid receptor (GR) gene. To do this we *de novo* sequenced the GR promoter region of DNA in hyenas. Given that there was no publicly available hyena genome at the time, we first made an in silico 'synthetic strand of DNA,' which was intended to represent our best guess at the nucleotide sequence for hyenas and which was based on the consensus base pairs from the alignment of multiple species' GR promoter sequences (Supplementary Figure 2). More specifically, our multiple species alignment contained human (including from McGowan *et al.* 2009), domestic dog, and walrus GR promoter DNA. Then moving across the alignment, we selected each nucleotide for our 'synthetic strand of DNA' at each position based on the greatest nucleotide similarity across

species or based on the nucleotide from species most closely related to hyenas when there was no clear consensus.

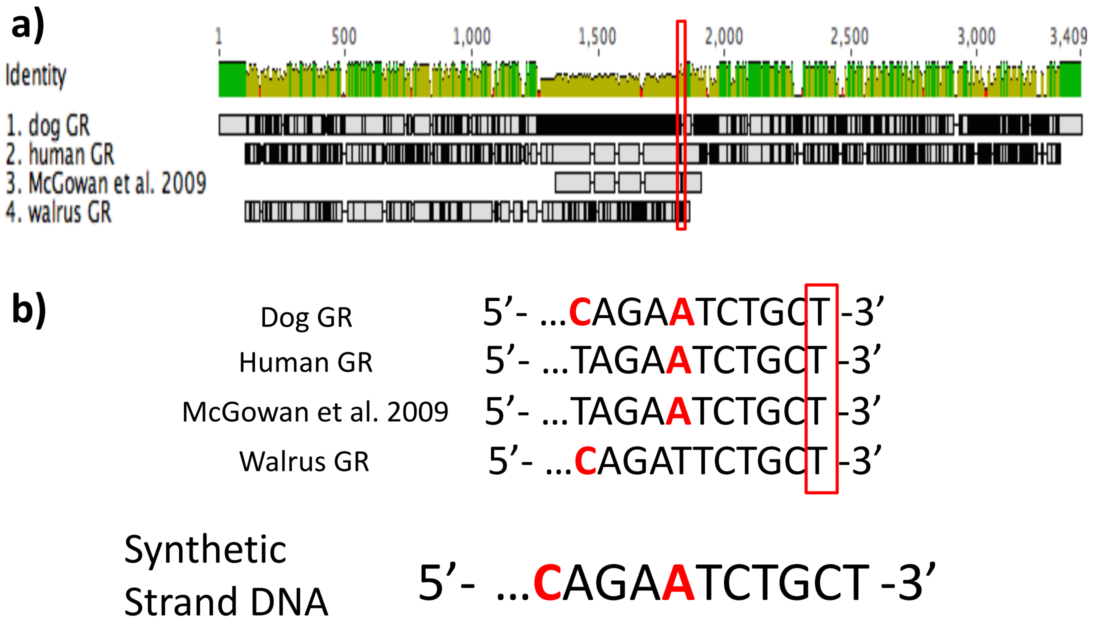

Supplementary Figure 2. a) A multiple species alignment of the promoter region of the Glucocorticoid Receptor, where consensus nucleotide identity is represented by the green bar at the top. b) A zoomed in view of the nucleotides from each species in the alignment and the 'synthetic strand of DNA' at the bottom. Red boxes represent the sliding window used to assess nucleotide consensus at each position and red nucleotides indicate a mismatch between one or more species.

Next, using our synthetic strand of DNA, we designed a tiling array of PCR primers with overlapping amplification products that targeted the GR promoter region, including parts of this promoter that had been shown to be differentially methylated in human and rodent studies (c.f. [20,21], Supplementary Table 2, and Supplementary Figure 3). We size selected spotted hyena DNA PCR products from gels based on the estimated amplicon lengths from our 'synthetic strand of DNA,' and Sanger sequenced the size matched products. In Sanger sequencing, we used genomic DNA extracted from whole blood samples taken from three individual hyenas.

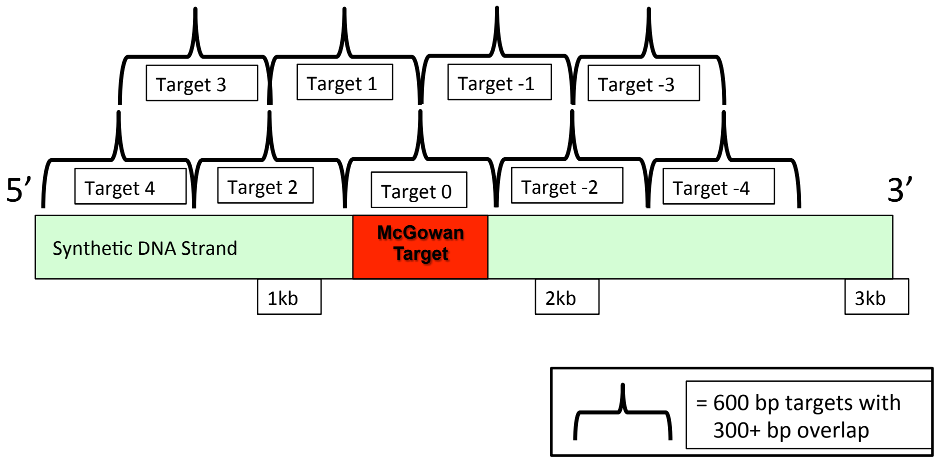

Supplementary Figure 3. Tiling array of PCR primers and targets aimed at generating overlapping DNA sequence from spotted hyenas that aligned with GR promoter region and included sequence of interest identified in human DNA methylation studies (e.g., McGowan *et al.* target).

Putative spotted hyena GR Promoter

5' - gggttctgctttgcaacttctctcccggtgggagagcgcgacggcggcgg
cggctgcagacgggg**cCGccc**agacgatgcggcggtgggggacctgccgg
cacgcgactacccccgagtcgagagtatgtacgcaccgaccccctcctct
ctcctcccctccctcagcctccccagagggcgtgtctggctgtccggccc
cgagcgaggccgagacgctgcggcaccgtttccttgcaaccctcgtagcc
ccgtgctaagtgacacagttcgcgcaactccgctccgaggcggcggcgcg
gaccactcccgcggccccgctgcgggccgcgccccggcctcccccaccc
cccacc**cCGccc**cagacagcccgctgtcacccgcaggggcgctgacggcg
ccggtcgccgagggactgggccagctccggagtgggtcgggagtcgcgcc
gctgggctg**gggCGg**g*aaggaggcagcgaggagagaaactagagaaactc*
*ggcttccctcccaagctcg*ccccggagagaccaggtcggcctccagccgc
cacctctccctttcccggagggtgggggg **- 3'**

Supplementary Figure 4. Spotted hyena putative GR promoter sequence based on Sanger sequencing of three individuals. The region of interest has been trimmed to match the aligned sequences published in Oberlander et al. 2008, McGowan et al. 2009, and Perroud et al. 2011. This sequence is 580 bp and has a 73.1% GC content. Underlined is the inferred consensus region (based on alignment) for human exon 1_F, and red, italicized is the inferred consensus region (based on alignment) for rat exon 1_7. GC boxes, the putative binding site for transcription factor NGFI-A, are **bold** and CpG sites are capitalized. The yellow highlighted GC box is the potential NGFI-A transcription binding site homologous to the region in rats that was shown to be differentially methylated with respect to maternal licking and grooming (c.f. Weaver et al. 2004).

We then aligned the overlapping hyena DNA reads from the Sanger sequencing and trimmed the hyena DNA to match published human GR DNA sequence [21–23], which resulted in the assembly of the putative GR promoter region for spotted hyenas (Supplementary Figure 4).

After sequencing the putative GR promoter in hyenas, we aligned our trimmed sequence with human and rat DNA sequences that were reported in the literature to be differentially methylated with respect to early-life adversity and maternal care, respectively [20–25] and as shown in Supplementary Figure 5. Focusing on an approximately 50 base pair region which overlapped with the differentially methylated regions in human and rat studies, including the putative transcription factor binding site for NGFI-A, we quantified CpG methylation using pyrosequencing on a Qiagen Pyromark® Q96 MD. We bisulfite treated genomic DNA extracted from whole blood, and then measured % DNA methylation in 78 hyena samples at 6 CpG sites. All primers used to generate PCR products for Sanger sequencing and the primers used in our pyrosequencing assay are listed in Supplementary Table 2.

Supplementary Table 2. A list of primers used in the candidate gene assays described in this study.

Purpose	Primer Name	Forward / Reverse / Sequencing	Primer Sequence	Nos. Base Pairs	Melting Temperature (°C)	GC%
PCR / Sanger sequencing *	GC_syn_2F	forward	GACGGTCGGAGCGCGGGGGAGGGTGGG	27	78.7	81.5
PCR / Sanger sequencing *	GC_syn_3F	forward	GGGACGGTCGGAGCGCGGGGGAGGGTGGG	29	80.9	82.8
PCR / Sanger sequencing *	GC_syn_4F	forward	CGGAGCTGGGCGGGGGCGGGAAGGAGGCA	29	81.1	79.3
PCR / Sanger sequencing *	syn_1F	forward	ACCGTTTCCGTGCAACCC	18	60.9	61.1
PCR / Sanger sequencing *	syn_3F	forward	GGAGGGTGGGTTCTGCTTT	19	59.5	57.9
PCR / Sanger sequencing *	syn_5F	forward	TGGGGGTTGAACTTGGCA	18	59	55.6
PCR / Sanger sequencing *	syn_6F	forward	ATTTGGCGTGCAACTTCCT	19	58.3	47.4
PCR / Sanger sequencing *	GC_syn_1R	reverse	CGCTCCCACTCCACCCCCGGCCGCTCCCC	29	81.3	82.8
PCR / Sanger sequencing *	syn_1R	reverse	TTCTCGCTGCCTCCTTCC	18	59	61.1
PCR / Sanger sequencing *	syn_3R	reverse	CAACCTGTTGGCGACGCT	18	61	61.1
PCR / Sanger sequencing *	syn_4R	reverse	GGGAACGGTGCAACCTGT	18	60.2	61.1
PCR / Sanger sequencing *	syn_6R	reverse	ACAGACTCGAGCTCGCAA	18	58.6	55.6
PCR / Sanger sequencing *	syn_7R	reverse	TTCCACCCACTAGAATCCGT	20	58.1	50
PCR / Sanger sequencing *	syn_8R	reverse	GTCTCCTTCCACCCACTAG	19	56.1	57.9
Pyrosequencing #	GR_meth_1.3_F	forward $	GAGGGATTGGGTTAGTTT	18	54.9	44.4
Pyrosequencing #	GR_meth_1.3_R	reverse	TTCTCTCCTCCCTACCTCCTTC	22	55.4	54.5
Pyrosequencing #	GR_meth_1.3_S	sequencing	CCTCCCTACCTCCTTCC	17	45.6	64.7

* Polymerase Chain Reaction (PCR) primers that were used to generate amplicons and in Sanger sequencing to construct the putative spotted hyena glucocorticoid receptor DNA sequence using N = 3 hyenas.

# Primers used in pyrosequencing to quantify percent CpG methylation in the putative GR promoter region among N = 78 hyenas.

$ Biotinylated primer for pyrosequencing.

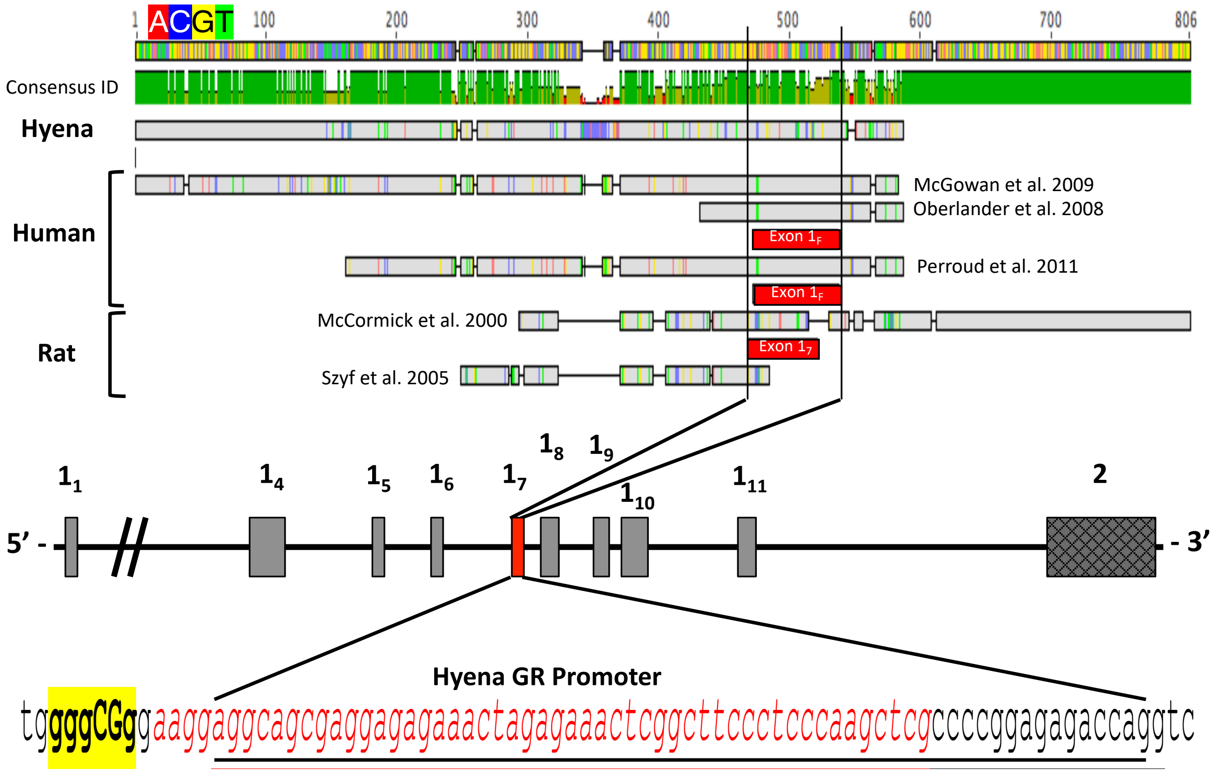

Supplementary Figure 5. Cross species comparison (spotted hyenas, humans and rats) of the GR promoter region that is the target of gene-specific DNA methylation studies. Base pair consensus from amplicons reported in multiple published studies as well as our spotted hyena amplicon are shown. Here we show hyena DNA sequence that corresponds with human exon 1_F, underlined and with rat exon 1_7, red and italicized. Also identified in yellow highlighted and bold text is the putative transcription factor binding site for NGFI-A.

Demographic, social experience, and ecological covariates

We estimated each hyena's age with an accuracy ± 7 days based on the behaviors and morphology of each cub when they were first observed outside of their natal den [26]. Once a hyena was three months old, we determined their sex based on the glans morphology of their erect phallus [27]. In this species, females are higher ranking, more aggressive, and larger than males, providing greater access to resources for them and their dependent offspring [28]. Maternal rank the year each offspring was born was calculated based on each adult female's wins and losses during agonistic interactions for a given year [29–31]. We normalized adult female

ranks each year on a scale of -1 (lowest rank) to 1 (highest rank) to account for changes in group size. We identified if young hyenas had a sibling or not and grouped them as twins or singletons, respectively. Using our detailed demographic data, we recorded each mom's parity for a given offspring and categorized this variable as either primiparous or multiparous. We also recorded clan size over the duration of our study period.

Next, we categorized offspring hyenas into groups according to whether they were born in low, medium, or high human disturbance based on illegal livestock grazing in the park [32]. We also grouped them into two categories of food availability based on the time of year that they were born. Birth dates roughly corresponding to the annual wildebeest and zebra migration from June – November were categorized as migration present, and hyenas born December – May were categorized as migration absent.

Statistical Analyses

Prior to formal analysis, we assessed the distribution of continuous variables as well as frequency tabulations of categorical variables to check for deviations from normality, errors in the data, missing values, and sample sizes within strata. We constructed boxplots to identify outliers and viewed scatterplots to check linearity of the associations between covariates and our outcome variables. We also examined bivariate associations between dependent and independent variables to help identify potential confounding variables that could influence the relationships of interest in our final models.

We organized our analyses into four parts and included two primary data sets for both global and genome-wide DNA methylation. The global DNA methylation data set used in our analyses comprises four overlapping data subsets with information on: (1) DNA methylation

measures from 186 cub and subadult hyenas (age ≤24 months), (2) maternal care behaviors from focal animal survey (FAS) data on 258 unique mother-cub pairs, (3) social network data from 115 hyenas during two early periods of development: the communal den (CD) period when a young hyena resided exclusively at the communal den, and the den independent (DI) period, which began when cubs were found away from the communal den on at least 4 consecutive occasions, and (4) fecal Glucocorticoid Metabolites (fGCMs) from 268 adult (≥24 months old) hyenas.

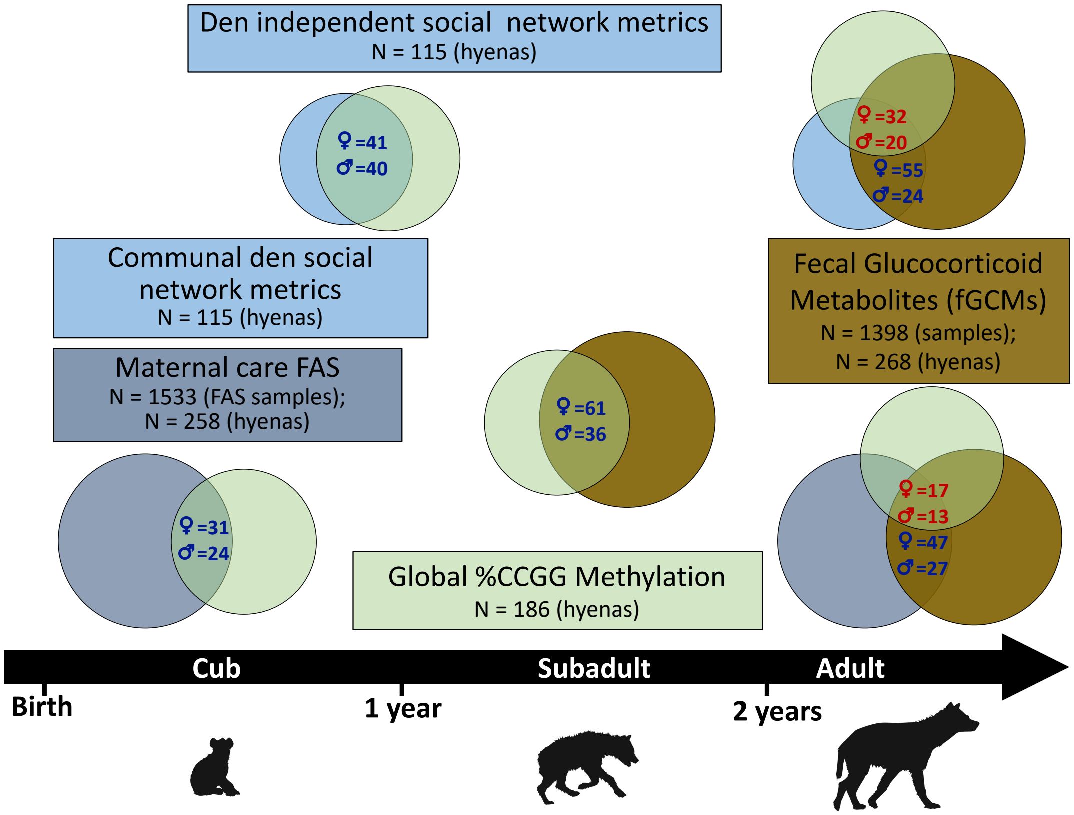

Supplementary Figure 6. Overview of the sampling design and sample sizes from the four data sets used in the analyses (Parts 1-3). The timeline on the bottom shows the different life stages of hyenas. Each color of box represents a distinct type of data collected from our study population, and the edges of boxes roughly correspond to the hyenas' ages when samples were collected. Overall sample sizes and numbers of individual hyenas are indicated inside each box. Venn diagrams show the overlap in sample size by sex among the different data sets and that correspond with the statistical models (purple = overlap of two data sets, and red = overlap of three data sets for mediation analyses).

The overlap among data sets and the final analytical samples sizes for analysis parts one through three are shown in Supplementary Figure 6. It is also worth noting that the earliest date of the maternal care FAS session and the start date of the social network period preceded the immobilization date when blood was drawn, and DNA methylation was measured. We also included only fGMCs that were obtained after assessments of maternal care behaviors, social network metrics, and %CCGG DNA methylation in order to preserve temporal relationships between our explanatory variables and our outcome variables to improve causal inference.

The fourth part of our analysis focused on our genome-wide DNA methylation data. We modeled associations between maternal rank, maternal care, DNA methylation and fGCMs using a two-step approach which is outlined in our conceptual diagram, Supplementary Figure 7. Using mERRBS, we assayed genome-wide DNA methylation among 29 hyenas, 25 of which also had fGCMs, and which additionally had information on maternal rank (n = 23) and maternal care metrics from FAS (n = 9). Similar to the maternal care metrics, we summarized repeated measures of fGCMs via a mixed-effects linear regression model in which the natural log of fGCMs was the dependent variable. We controlled for each hyena's age in months, reproductive state, and time of day (AM vs PM) when the fecal sample was collected, a random intercept for offspring ID to account for correlations between

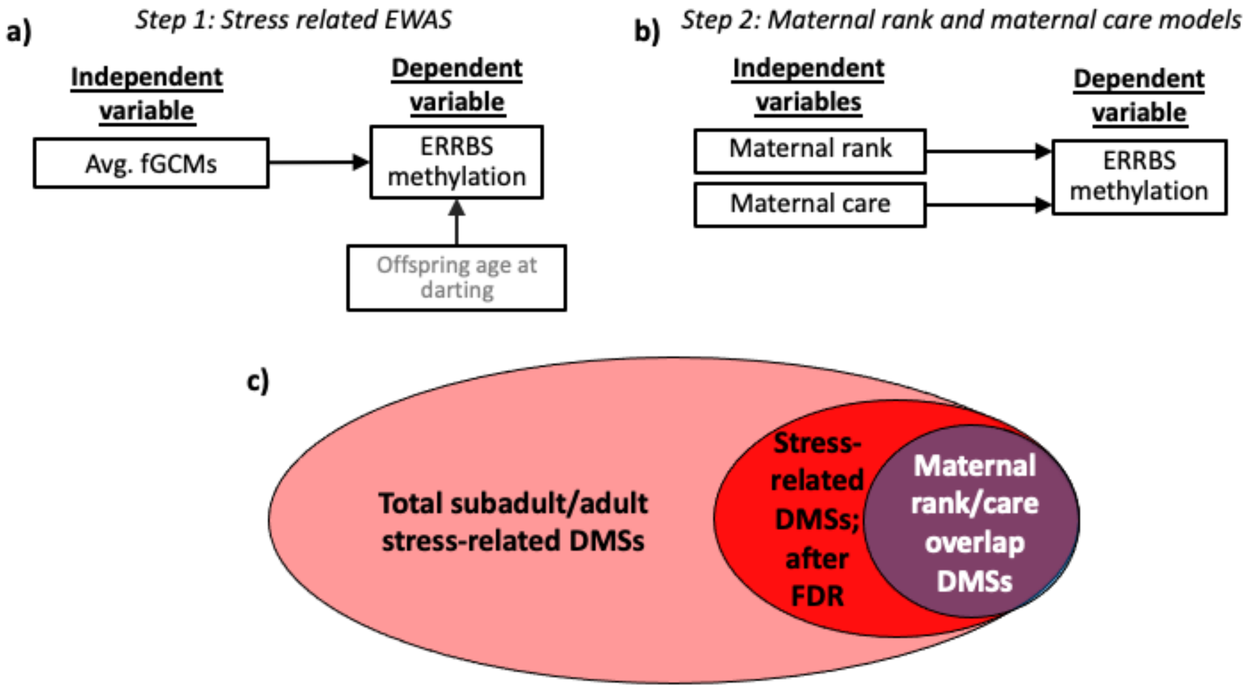

Supplementary Figure 7. Conceptual diagram a) of the epigenome-wide association study (EWAS) for identification of differentially methylated sites (DMSs) from enhanced reduced representation bisulfite sequencing (mERRBS) data that are associated with subadult/adult fecal Glucocorticoid Metabolites (fGCMs) BLUPs, b) conceptual models of maternal rank and maternal care associations with DMSs identified in step 1, c) and a Venn diagram of potentially mediating stress-related DMSs that passed the false discovery rate (FDR) correction (bright red) and were also associated with maternal rank/care variables (in purple).

samples collected from the same individual, and an unstructured covariance matrix. Repeated fGCMs were limited to when hyenas were 1 yr or older so that our assessment of the stress phenotype roughly overlapped or proceeded the assessment of genome-wide DNA methylation. We then calculated BLUPS from this mixed-effects model. The BLUPs effectively represent each hyena's deviation in fGCMs relative to the population average after accounting for key demographic covariates. This method has been previously used to consolidate repeated measurements of a given variable into a single value per individual without making assumptions about the underlying distribution of the data [33,34]. Linear mixed-models were run using the R package lme4 [35].

Prior to conducting our epigenome wide association study (EWAS), we filtered the mERRBS data set. In order to reduce the type I error rate and the burden of multiple comparisons correction, we removed 5% of CpG sites that had the lowest inter-individual variation of DNA methylation [36]. We also excluded CpG sites with low (<10%) and high (>90%) average DNA methylation. While this filtering should increase the signal to noise ratio in our analysis, there is a trade off in which some true but small effect size associations may not be captured. We note this limitation given that we and others have reported and discussed the biological relevance of small differences in DNA methylation, particularly in developmental origins studies [37,38].

We accounted for relatedness among individuals comprising our study population by including a genetic covariance matrix in our EWAS models. We built the genetic covariance matrix by combining our extensive hyena lineage data [7] with paternity data determined from an established method utilizing microsatellites from DNA extracted from blood and fecal samples [39]. We determined paternity for 24 of 29 individuals and maternal ID was known for all study animals. With these data we constructed a pedigree and used the R package, 'AGHmatrix,' [40] to calculate a relatedness A-matrix, which we used to control for genetic covariance in our EWAS.

After conducting our EWAS we annotated each analyzed CpG site in the hyena genome following the same method as previously described for mapping the 'CCGG' motif. Among CpG sites that were differentially methylated in our EWAS, we used the web-based BLAST-like alignment tool (BLAT) [41] tool from UCSC Genome Browser to map the region surrounding each DMS to the domestic cat (*Felis catus* Nov. 2017 [felCat9] Assembly) and human (UCSC Human Dec. 2010 [GRCh38/hg38] Assembly) genomes. Using the default settings, BLAT searches whole reference genomes using an 11-mer tile size and a step size of 5 to identify homologous regions

between the query sequence of DNA and the reference genome [41]. We report hits in which the hyena DNA sequence queried against cat and human genomes showed agreement, and where the top BLAT score for matched alignments is > 500 and the percent identical match are > 85%.

Supplementary Results

Analysis parts 1-3 background characteristics and descriptive statistics

Mother and offspring hyena pairs spent a mean ± SD of the proportion of time they were observed together during FAS sessions in close proximity (0.826 ± 0.099), nursing (0.458 ± 0.064), and grooming (0.073 ± 0.052). During the CD period of development, the mean ± SD of association index social network metrics were 57.45 ± 13.29 for degree centrality, 5.85 ± 2.11 for strength, and 6.50 ± 11.81 for betweenness centrality. During the DI period, the mean ± SD for degree, strength, and betweenness were 61.59 ± 15.94, 4.18 ± 1.54, and 6.79 ± 6.11, respectively. We estimated a mean ± SD of fGCMs of 104.03 ± 95.93 ng/g for 268 hyenas with at least one adult fecal sample (we averaged repeated measure for an individual). Finally, we calculated a mean ± SD of 75.53 ± 3.03 %CCGG methylation for the 186 hyena cubs and subadults in our study population. We annotated the 'CCGG' motif, which is targeted by LUMA, and confirmed that the majority of 'CCGG' motifs are found in intergenic regions and introns of the hyena genome (Supplementary Figure 8). More specifically we identified 2,191,145 'CCGG' motifs of which roughly 63% were in intergenic regions and 29% in introns, while only 5% were in exons and 3% in promoters. Previous work estimated that there are approximately 2.4 million CCGG motifs in the typical mammalian genome, and based upon the high resolution human genome, about 3% of the 'CCGG' motifs occur within 1kb of transcription start sites, 45% in gene bodies and 52% in non-coding regions [11,42,43].

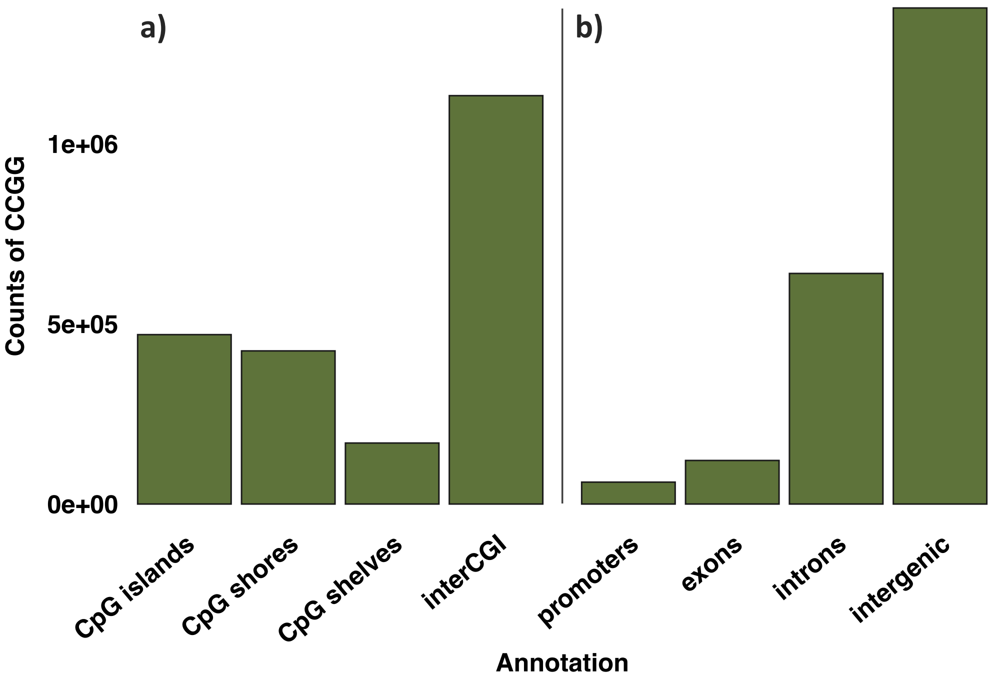

Supplementary Figure 8. The 'CCGG' motif, which is targeted by the LUminometric Methylation Assay (LUMA), annotated in the spotted hyena genome. a) Annotation categories are based on CpG density using the UCSC genome tool 'cpg_lh'. CpG shores include the region of the genome 2kb up and downstream of the CpG island start and end boundaries. CpG shelves include the region of the genome 2kb up and downstream of the CpG shore start and end boundaries. The interCGI region includes the remaining hyena genome. b) Genic annotations are based on the draft hyena genome and the general feature format (GFF) file. Annotated counts add up to more than 2,191,145 unique 'CCGG' motifs due to this 4-bp motif overlapping annotation boundaries.

Bivariate analysis: Global (%CCGG) DNA methylation data set

In bivariate analyses, we assessed crude associations between potential confounding variables with each variable of interest, namely maternal care FAS behaviors, association social network metrics and fGCMs. Many of these associations have been published previously [5,44], so we do not interpret or discuss bivariate results, but rather report the model estimates in Supplementary Tables 3-6. Variables associated with %CCGG methylation have recently been reported [11], so we do not show those results here.

Supplementary Table 3. Bivariate associations of demographic, early-life social experience, and ecological covariates with maternal care behavior BLUPs among N = 258 mother-offspring pairs. P-values are based on a T-statistic from a two-sided test.

	β (± SE)†					
	Close proximity	*P*	Nursing	*P*	Grooming	*P*
Biological confounding variables						
Sex						
Female	0.00 (Reference)		0.00 (Reference)		0.00 (Reference)	
Male	-0.01 (± 0.01)	0.497	0.00 (± 0.01)	0.603	0.01 (± 0.01)	0.069
Unknown	0.01 (± 0.03)	0.707	-0.02 (± 0.02)	0.409	0.02 (± 0.02)	0.145
Social experience confounding variables						
Number of litter mates						
Singleton	0.00 (Reference)		0.00 (Reference)		0.00 (Reference)	
Twins	0.04 (± 0.01)	0.014	0.02 (± 0.01)	0.011	-0.02 (± 0.01)	0.002
Mother's parity						
Primiparous	0.00 (Reference)		0.00 (Reference)		0.00 (Reference)	
Multiparous	-0.02 (± 0.01)	0.106	0.01 (± 0.01)	0.142	0.00 (± 0.01)	0.793
Ecological confounding variables						
Human disturbance (year offspring born)						
Low	0.00 (Reference)		0.00 (Reference)		0.00 (Reference)	
Medium	-0.02 (± 0.02)	0.125	-0.01 (± 0.01)	0.141	-0.01 (± 0.01)	0.104
High	0.05 (± 0.02)	0.002	0.05 (± 0.01)	<0.001	0.01 (± 0.01)	0.401
Migration status on date of birth						
Migration absent	0.00 (Reference)		0.00 (Reference)		0.00 (Reference)	
Migration present	-0.02 (± 0.01)	0.219	-0.02 (± 0.01)	0.003	0.00 (± 0.01)	0.454

†Estimates represent differences in incident rates or proportions of time spent engaged in a particular behavior in relation to the total time the mother-offspring pair were observed together (n = 258 pairs).

Supplementary Table 4. Bivariate associations of demographic, early-life social experience, and ecological covariates with communal den (CD) social network metrics among N= 115 hyenas. P-values are based on a T-statistic from a two-sided test.

	β (± SE)†					
	Degree†	*P*	Strength	*P*	Betweeness	*P*
Biological confounding variables						
Sex						
Female	0.00 (Reference)		0.00 (Reference)		0.00 (Reference)	
Male	3.98 (± 2.49)	0.113	0.60 (± 0.39)	0.132	3.84 (± 2.21)	0.085
Social experience confounding variables						
Number of litter mates						
Singleton	0.00 (Reference)		0.00 (Reference)		0.00 (Reference)	
Twins	-1.39 (± 4.03)	0.744	0.47 (± 0.63)	0.460	-3.26 (± 1.89)	0.087
Clan size						
Number of hyenas in clan	0.54 (± 0.10)	<0.001	0.03 (± 0.02)	0.059	0.23 (± 0.10)	0.020
Ecological confounding variables						
Human disturbance (year offspring born)						
Low	0.00 (Reference)		0.00 (Reference)		0.00 (Reference)	
Medium	-8.57 (± 2.66)	<0.001	-0.37 (± 0.41)	0.369	3.75 (± 2.46)	0.130
High	12.41 (± 2.79)	<0.001	1.82 (± 0.50)	<0.001	-0.07 (± 3.03)	0.981
Migration status on date of birth						
Migration absent	0.00 (Reference)		0.00 (Reference)		0.00 (Reference)	
Migration present	-5.96 (± 2.57)	0.022	-1.64 (± 0.39)	<0.001	-1.36 (± 2.33)	0.560

†Estimates are based on association index networks from 115 communal den dependent hyeans.

Supplementary Table 5. Bivariate associations of demographic, early-life social experience, and ecological covariates with DI social network metrics among N = 115 hyenas. P-values are based on a T-statistic from a two-sided test.

	β (± SE)[†]					
	Degree	*P*	Strength	*P*	Betweeness	*P*
Biological confounding variables						
Sex						
Female	0.00 (Reference)		0.00 (Reference)		0.00 (Reference)	
Male	2.70 (± 3.01)	0.371	0.28 (± 0.29)	0.332	0.17 (± 1.16)	0.884
Social experience confounding variables						
Number of litter mates						
Singleton	0.00 (Reference)		0.00 (Reference)		0.00 (Reference)	
Twins	5.15 (± 4.86)	0.292	0.80 (± 0.46)	0.086	-1.30 (± 1.66)	0.435
Clan size						
Number of hyenas in clan	0.77 (± 0.08)	<0.001	0.03 (± 0.01)	0.014	0.13 (± 0.04)	0.002
Ecological confounding variables						
Human disturbance (year offspring born)						
Low	0.00 (Reference)		0.00 (Reference)		0.00 (Reference)	
Medium	-12.29 (± 2.59)	<0.001	-0.93 (± 0.30)	0.002	-1.22 (± 1.28)	0.345
High	14.75 (± 3.20)	<0.001	0.70 (± 0.36)	0.058	-0.66 (± 1.58)	0.676
Migration status on date of birth						
Migration absent	0.00 (Reference)		0.00 (Reference)		0.00 (Reference)	
Migration present	-0.82 (± 3.15)	0.796	0.33 (± 0.30)	0.282	-1.83 (± 1.20)	0.130

[†]Estimates are based on association index networks from 115 communal den independent hyeans.

Supplementary Table 6. Bivariate associations of sample collection variables and adult fecal Glucocorticoid Metabolites (fGCMs) based on N = 1398 samples collected from N = 268 adult hyenas. P-values are based on a T-statistic from a two-sided test.

	β (± SE)[†]	
Potential confounding variables	fGCMs	*P*
Sex		
Female	0.00 (Reference)	
Male	-0.72 (± 0.10)	<0.001
Standardized Maternal Rank (yr offspring born)	-0.13 (± 0.09)	0.150
Human disturbance (yr offspring born)		
Low	0.00 (Reference)	
Medium	0.24 (± 0.08)	0.004
High	-0.08 (± 0.09)	0.366
Precision covariates (assessed on fecal collection date)		
Age (months)	0.005 (± 0.001)	<0.001
Reproductive state		
Nulliparous	0.00 (Reference)	
Pregnant	0.98 (± 0.11)	<0.001
Lactating	0.59 (± 0.09)	<0.001
Other	0.65 (± 0.11)	<0.001
Male	-0.18 (± 0.12)	0.133
Time of day		
AM	0.00 (Reference)	
PM	-0.50 (± 0.05)	<0.001
Migration status		
Migration absent	0.00 (Reference)	
Migration present	0.10 (± 0.06)	0.091

[†]Beta estimate are differences in each categorical variable from the reference group in fGCMs (ng/g) on the natural log scale from mixed models in which hyena ID was included as a random intercept. Models include 1398 fGCMs samples collected from 268 adult (≥24 months old) hyenas.

Mediation analyses

Supplementary Table 7. Association of maternal care and early-life social network metrics with %CCGG methylation and adult fecal Glucocorticoid Metabolites (fGCMs) among N = 30 (matneral care models) or N = 52 hyenas (social network models). Summary of results from steps 1-2 of a mediation analysis. *X* represents the explanatory variable (early-life social experience), *M* represents the potential mediator (%CCGG methylation), and *Y* represents the outcome (fGCMs).

	N	β (95% CI)			
		Step 1: *X* → *Y* [†]	Step 2a: *X* → *M* [‡]		Step 2b: *M* → *Y* [†]
Maternal Care FAS (per 1-SD)				DNA Methylation (per 1-SD) [§]	
Close proximity	30	-0.08 (-0.38, 0.21)	1.45 (0.62, 2.26)	%CCGG	-0.02 (-0.36, 0.33)
Nursing	30	-0.07 (-0.43, 0.31)	0.52 (-0.73, 1.82)		
Grooming	30	-0.16 (-0.43, 0.10)	0.80 (-0.49, 2.09)		
CD period (per 1-SD)					
Degree	52	-0.08 (-0.24, 0.09)	0.17 (-0.87, 1.20)		
Strength	52	0.01 (-0.15, 0.18)	0.31 (-0.61, 1.23)		
Betweenness	52	-0.10 (-0.33, 0.13)	-0.13 (-0.91, 0.66)	DNA Methylation (per 1-SD) [ϕ]	
DI period (per 1-SD)				%CCGG	0.06 (-0.13, 0.24)
Degree	52	-0.11 (-0.27, 0.06)	0.18 (-1.29, 1.65)		
Strength	52	-0.08 (-0.24, 0.08)	0.61 (-0.32, 1.54)		
Betweenness	52	-0.06 (-0.22, 0.10)	-0.17 (-1.01, 0.68)		

[†]Beta estimate are fGCMs (ng/g) on the natural log scale from mixed models in which hyena ID was included as a random intercept. Models are adjusted for hyena's age (months), sex, reproductive state among females, the time of day when the fecal sample was collected and human disturbance in the birth yr.

[‡] Models are adjusted for hyena's age when blood was drawn (months), sex, number of littermates, maternal rank, mom's parity / clan size, human disturbance in the birth yr, and the migration status on the birth date.

[§] Assocation is based on samples for which there are measures of maternal care, DNA methylation, and fGCMs.

[ϕ] Assocation is based on samples for which there are measures of social connectedness during the CD and DI periods, DNA methylation, and fGCMs.

Bolded values indicate statistical significance at alpha = 0.05.

Genome-wide (mERRBS) DNA methylation analyses

We analyzed data from 29 female hyenas which had an average ± SD age of 18.8 ± 3.9 months on the date their DNA methylation was assessed. After processing the data through the bioinformatic pipeline, we quantified DNA methylation at 2,243,867 unique CpG sites that had a minimum of 10x coverage in all 29 hyenas. The average bisulfite conversion rate was 99.4% (SD ± 0.1%) and the average alignment efficiency of reads to the draft spotted hyena genome was 75.5% (SD ± 2.6) for the 29 hyena samples. After removing low variation as well as high and low average DNA methylation CpG sites from the mERRBS data, we retained 663,926 loci which

were included in our EWAS. Of the 29 hyenas for which we had mERRBS data, we also had fecal samples from 25 of them (median of 3 samples per hyena). Mean ± SD later life corticosterone concentration in our study population was 72.9 ± 62.8 ng/g. During the FAS data collection, we observed mother-offspring pairs together on average for 26 minutes per FAS session, with a median of 2.5 sessions recorded per pair. In this subsample, mothers spent an average of 79.8% (SD ± 26.0%) of their time in close proximity to offspring, 66.4% (SD ± 31.2%) of time nursing, and 4.9% (± 7.4%) of time grooming.

We graphed the P-value distribution (Supplementary Figure 9, a) and a Q-Q plot of the expected vs. the observed P-values (Supplementary Figure 9, b) from our uncorrected EWAS. These plots indicated the presence of test statistic bias and inflation, likely due to unmeasured confounding, so we used the package 'bacon' to improve estimation of the empirical null distribution [45]. After applying the 'bacon' correction we no longer observed depletion of low P-values (Supplementary Figure 9).

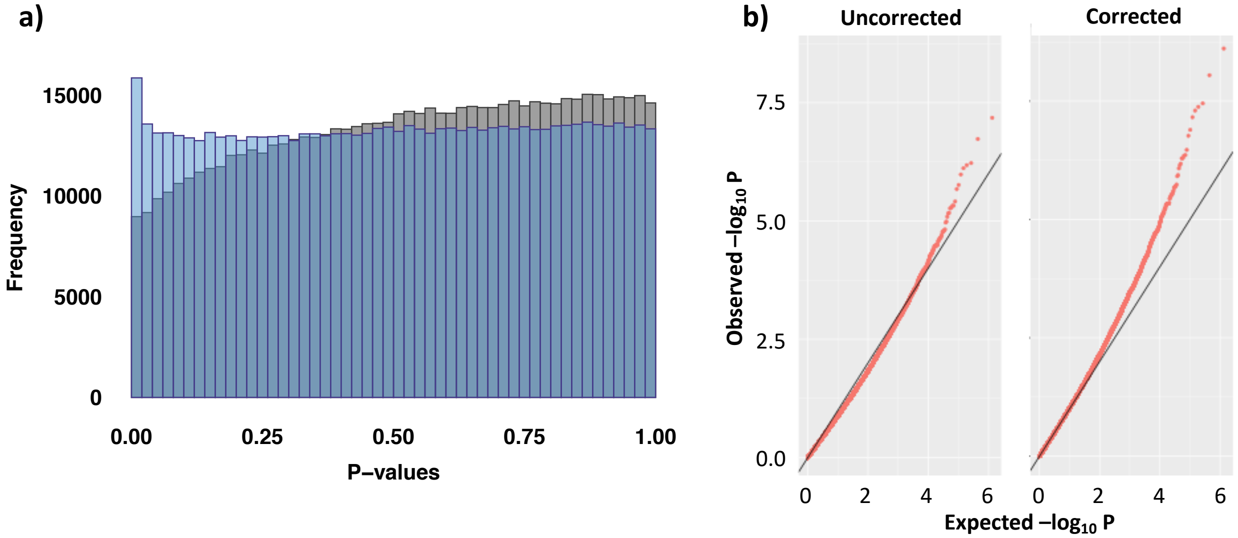

Supplementary Figure 9. Diagnostic plots for stress related EWAS among N = 25 hyenas. a) A frequency histogram showing the distribution of uncorrected P-values (gray bars) and bacon corrected P-values (blue bars) generated from the EWAS. b) QQ-plots showing the observed $-\log_{10}$ P-values versus the expected $-\log_{10}$ P-values for the uncorrected and bacon corrected P-values.

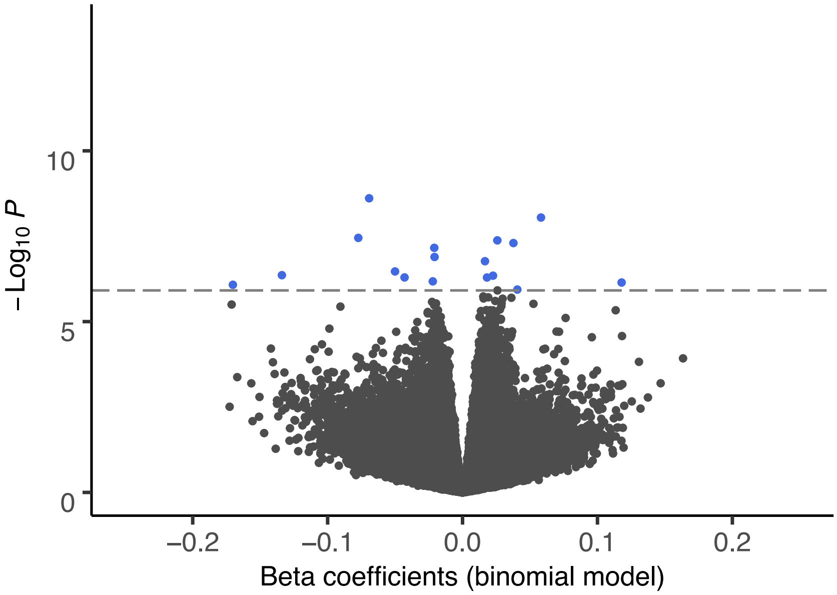

Supplementary Figure 10. Volcano plot of the $-\log_{10}$ p-values by the beta coefficients from a binomial regression epigenome wide association study (EWAS) among N = 25 hyenas after bias and inflation correction using the bacon package in R. Counts of methylated over total cytosines from enhanced reduced representation bisulfite sequencing data were modeled as a function of variation in subadult and adult spotted hyena fecal Glucocorticoid Metabolites (fGCMs). The horizontal line represents the threshold for Benjamini-Hochberg false discovery rate (FDR) cutoff of 5%, above which the blue points are significant.

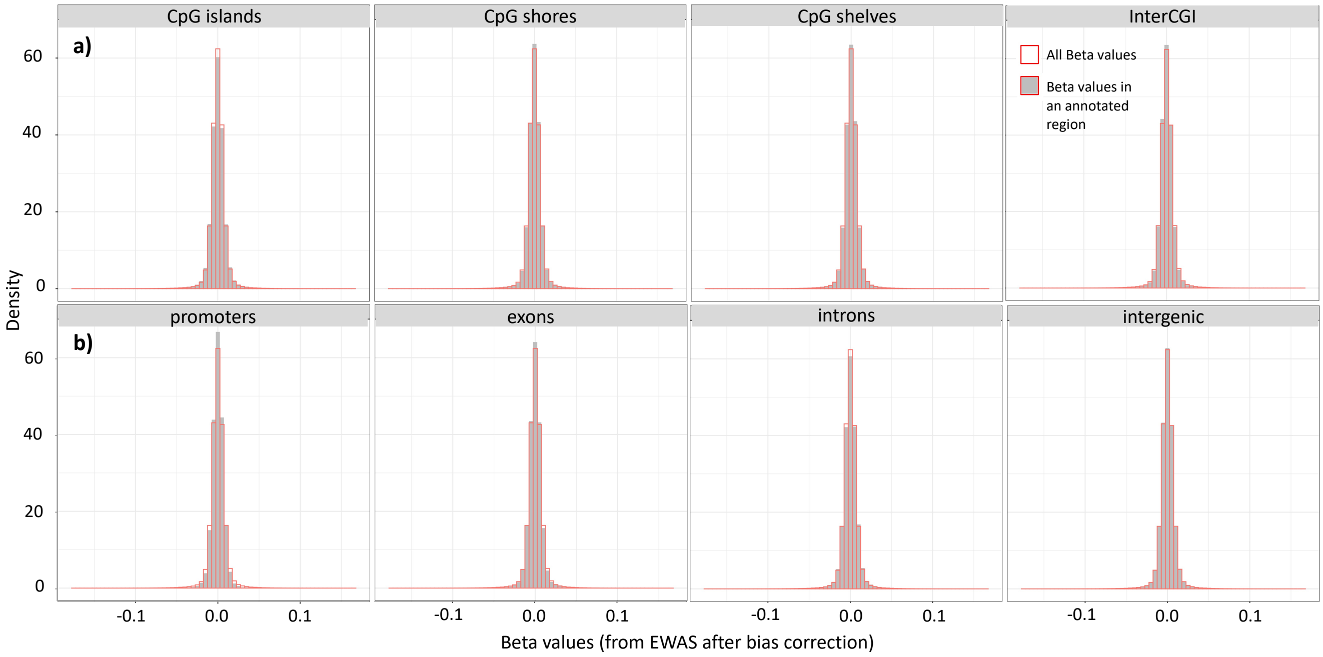

Supplementary Figure 11. Density plots of Beta values from the stress hormone EWAS after bias and inflation correction among N = 25 hyenas. All Beta values (white histogram) are overlaid with Beta values from specific annotation regions (gray filled histogram). a) Beta values from stress hormone EWAS based on the CpG density annotation. b) Beta values from stress hormone EWAS based on genic annotation.

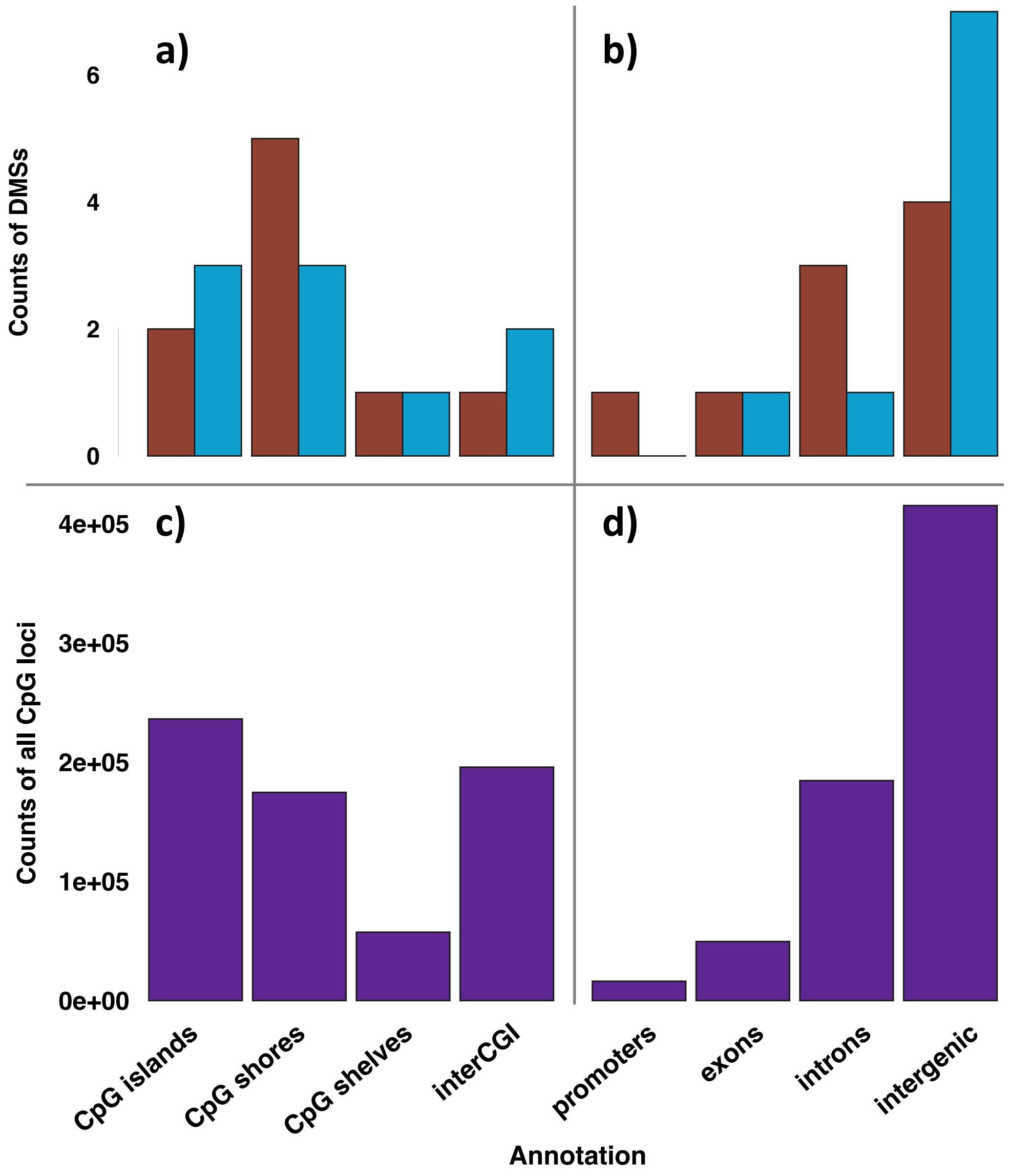

Supplementary Figure 12. Annotated counts of CpG sites identified from the stress hormone EWAS among N = 25 hyenas. a) Counts of differentially methylated sites (red bars = positive associations; blue bars = negative associations) annotated based on CpG density from the spotted hyena genome using the UCSC genome tool 'cpg_lh'. CpG shores include the region of the genome 2kb up or downstream of the CpG island start and end boundaries. CpG shelves include the region of the genome 2kb up or downstream of the CpG shore start and end boundaries. The interCGI region includes the remaining hyena genome. b) Counts of differentially methylated sites summarized by genic annotations, which are based on the draft hyena genome and the general feature format (GFF) file. In both panels a) and b), significant DMSs are determined after P-values are bias and inflation corrected and controlled for multiple comparison using Benjamini-Hochberg false discovery rate (FDR) of 5%. c) Annotated counts of all CpG loci tested in the stress hormone EWAS based on the CpG density annotation. d) Annotated counts of all CpG loci tested in the stress hormone EWAS based on genic annotation.

Supplementary Table 8. Epigenome wide association results showing differentially methylated CpG sites (DMSs) associated with fecal Glucocorticoid Metabolites (fGCMs) Best Linear Unbiased Predictors (BLUPs) among N = 25 hyenas. A partial list of DMSs excluding those significantly associated with a maternal care metric at P<0.1, which are reported in the main test. DNA methylation was assessed during the subadult life stage, and fecal samples were collected from animals during the subadult and adult life stages.

CpG location in *C. crocuta* genome (scaffold #)	β ± SE †	FDR P-value ‡	*P* †	Nearest human gene(s)§	Approx. location ¶	CHR¶	Human Ref. BLAT score/% identical match/# bp span §	Cat Ref. BLAT score/% identical match/# bp span ↞	Gene name §
119.9561382	-0.021 ± 0.004	0.007653	6.92E-08	*MROH5/TSNARE1*	non-coding	8	1224/86.0%/15562	10520/88.5%/20751	*maestro heat like repeat family member 5 / T-SNARE domain containing 1*
124.5690667	-0.050 ± 0.010	0.025045	3.40E-07	NA	NA	NA	385/81.0%/1058	12372/89.3%/24937	NA
221.28127	0.017 ± 0.003	0.01414	1.70E-07	NA	NA	NA	260/74.3%/733	1173/86.5%/2147	NA
234.3124311	0.041 ± 0.008	0.045	1.15E-06	*MAPK8IP3/JPT2*	gene body	16	612/88.3%/7972	8897/87.1%/20052	*mitogen-activated protein kinase 8 interacting protein 3 /Jupiter microtubule associated homolog 2*
273.4081006	-0.170 ± 0.035	0.034618	8.34E-07	*RADIL*	gene body	7	1202/87.4%/44037	8010/87.1%/26763	*ras-associating and dilute domain-containing protein*
28.1531871	0.026 ± 0.005	0.00665	4.18E-08	*ZMIZ1*	non-coding (>500kb)	10	3486/86.1%/19445	13148/89.3%/19547	*zinc finger MIZ-type containing 1*
32.11390064	0.058 ± 0.010	0.00298	8.97E-09	*PITRM1*	non-coding (<20kb)	10	547/86.1%/28073	7291/86.3%/19469	*pitrilysin metallopeptidase 1, transcript variant 2*
334.155724	-0.077 ± 0.014	0.006652	3.53E-08	*MYBPC2*	gene body	19	3622/87.7%/24496	12434/89.9%/22344	*myosin binding protein C, fast type*
415.2139465	-0.134 ± 0.027	0.026112	4.37E-07	*CRNN*	gene body	1	2754/85.3%/28728	14095/90.2%/21398	*cornulin*
443.367667	-0.043 ± 0.009	0.026112	5.09E-07	*PLPPR3/AZU1/PRTN3/ELANE*	gene body	19	1727/88.5%/42763	7967/89.1%/24540	*phospholipid phosphatase related 3 /azurocidin 1/proteinase 3/elastase, neutrophil expressed*
53.1319257	-0.069 ± 0.012	0.001627	2.45E-09	*MACROD1/FLRT1*	gene body	11	3395/88.1%/20543	13137/89.8%/20758	*mono-ADP ribosylhydrolase 1/fibronectin leucine rich transmembrane protein 1*
64.5896792	-0.021 ± 0.004	0.012154	1.28E-07	*SOWAHA/SHROOM1*	gene body	5	6198/86.1%/26199	13935/90.8%/20319	*sosondowah ankyrin repeat domain family member A/shroom family member 1*

† Beta estimates and unajusted p-values are from binomial regression models run in program MACAU and then corrected for inflation and bias using the package bacon.

‡ FDR p-value based on Benjamini-Hochberg correction (5%)

§ Mapping of spotted hyena DNA sequences spanning 20.4k bp onto the human genome (Human Dec. 2013 [GRCh38/hg38] Assembly) was done using the top hit from the UCSC Genome Browser's BLAT tool.

↞ Mapping of spotted hyena DNA sequences spanning 20.4k bp onto the domestic cat genome (*Felis catus* Nov. 2017 [felCat9] Assembly) was done using the top hit from the UCSC Genome Browser's BLAT tool.

¶ Reference genome is hg38

Abbreviations: CHR - chromosome

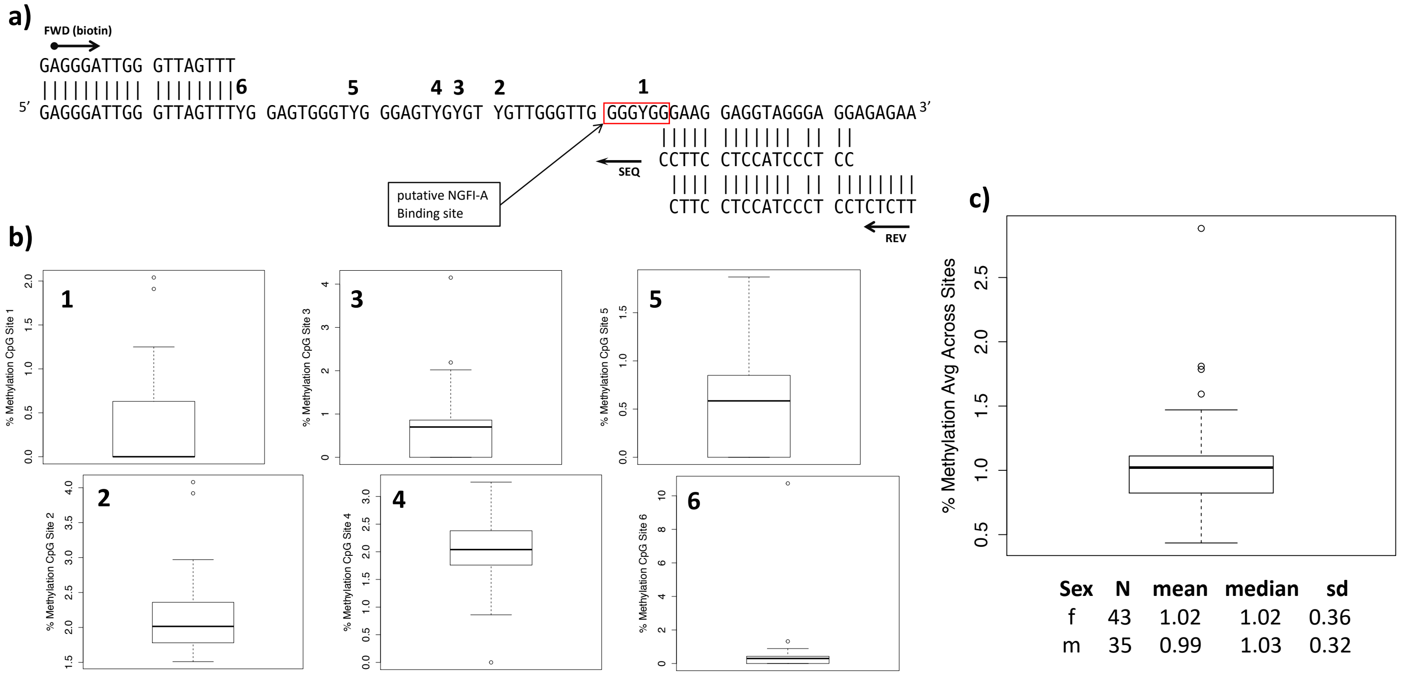

Sex	N	mean	median	sd
f	43	1.02	1.02	0.36
m	35	0.99	1.03	0.32

Supplementary Figure 13. Quantitative measurement of DNA methylation at 6 CpG sites in the hyena putative GR promoter based on a DNA sequence that was targeted in previous rat and human studies. All box and whisker plots represent the 25th percentile (Q1) the 50th percentile (media), the 75th percentile (Q3) of the data. Whiskers are based on the interquartile range (IQR) such that the lower whisker represents Q1 – IQR * 1.5, and the upper whisker represents Q3 + IQR * 1.5. a) Target DNA sequence with potentially methylated cytosine bases identified with a 'Y' and numbered according to pyrosequencing order. Putative transcription factor binding site is identified in the red box and forward, reverse and sequencing primers are shown on the DNA strand. b) Boxplots showing site-specific CpG percent methylation. c) Boxplot and descriptive statistics showing percent methylation values averaged across all 6 CpG sites from N = 78 hyenas (note some samples did not pass the pyrosequencing quality standards, so the total samples size dropped from 93 hyenas).

Supplementary References

1. Holekamp, K. E. & Smale, L. Behavioral Development in the Spotted Hyena. *Bioscience* **48**, 997–1005 (1998).
2. Brooks, M. E. *et al.* glmmTMB balances speed and flexibility among packages for zero-inflated generalized linear mixed modeling. *R J.* **9**, 378–400 (2017).
3. Magnusson, A. *et al.* glmmTMB: Generalized Linear Mixed Models using Template Model Builder. 1–32 (2019).
4. Cameron, A. C. & Trivedi, P. K. *Regression analysis of count data*. (Cambridge University Press, 1998).
5. Turner, J. W., Bills, P. S. & Holekamp, K. E. Ontogenetic change in determinants of social network position in the spotted hyena. *Behav. Ecol. Sociobiol.* **72**, (2018).
6. Cairns, S. J. & Schwager, S. J. A comparison of association indices. *Anim. Behav.* **35**, 1454–1469 (1986).
7. Holekamp, K. E., Smith, J. E., Strelioff, C. C., Van Horn, R. C. & Watts, H. E. Society, demography and genetic structure in the spotted hyena. *Mol. Ecol.* **21**, 613–632 (2012).
8. Wey, T. W. & Blumstein, D. T. Social attributes and associated performance measures in marmots: bigger male bullies and weakly affiliating females have higher annual reproductive success. *Behav. Ecol. Sociobiol.* **66**, 1075–1085 (2012).
9. Karimi, M., Johansson, S. & Ekström, T. J. Using LUMA. A Luminometric-Based Assay for Global DNA Methylation. *Epigenetics* **1**, 45–48 (2006).
10. Karimi, M. *et al.* LUMA (LUminometric Methylation Assay)--a high throughput method to the analysis of genomic DNA methylation. *Exp. Cell Res.* **312**, 1989–1995 (2006).
11. Laubach, Z. M. *et al.* Early life social and ecological determinants of global DNA methylation in wild spotted hyenas. *Mol. Ecol.* **28**, 3799–3812 (2019).
12. Kent, W. J. UCSC Genome Browser. (2020).
13. Cavalcante, R. G. & Sartor, M. A. annotatr: genomic regions in context. *Bioinformatics* (2017). doi:10.18129/B9.bioc.annotatr
14. Yang, C. *et al.* A draft genome assembly of spotted hyena, Crocuta crocuta. *Sci. Data* **7**, 1–10 (2020).
15. Gu, H. *et al.* Preparation of reduced representation bisulfite sequencing libraries for genome-scale DNA methylation profiling. *Nat. Protoc.* **6**, 468–481 (2011).
16. Garrett-Bakelman, F. E. *et al.* Enhanced Reduced Representation Bisulfite Sequencing for assessment of DNA nethylation at base pair resolution. *J. Vis. Exp.* 1–15 (2015). doi:10.3791/52246
17. Krueger, F. & Andrews, S. R. Bismark: A flexible aligner and methylation caller for Bisulfite-Seq applications. *Bioinformatics* **27**, 1571–1572 (2011).
18. Langmead, B. & Salzberg, S. L. Fast gapped-read alignment with Bowtie 2. *Nat. Methods* **9**, 357–359 (2012).
19. Akalin, A. *et al.* MethylKit: a comprehensive R package for the analysis of genome-wide DNA methylation profiles. *Genome Biol.* **13**, R87 (2012).
20. Weaver, I. C. G. *et al.* Epigenetic programming by maternal behavior. *Nat. Neurosci.* **7**, 847–854 (2004).
21. McGowan, P. O. *et al.* Epigenetic regulation of the glucocorticoid receptor in human brain associates with childhood abuse. *Nat. Neurosci.* **12**, 342–348 (2009).
22. Oberlander, T. F. *et al.* Prenatal exposure to maternal depression, neonatal methylation

of human glucocorticoid receptor gene (NR3C1) and infant cortisol stress responses. *Epigentics* **3**, 97–106 (2008).
23. Perroud, N. *et al.* Increased methylation of glucocorticoid receptor gene (NR3C1) in adults with a history of childhood maltreatment: a link with the severity and type of trauma. *Transl. Psychiatry* **1**, e59 (2011).
24. Mccormick, J. A. *et al.* 5'-Heterogeneity of Glucocorticoid Receptor Messenger RNA Is Tissue Specific : Differential Regulation of Variant Transcripts by Early-Life Events. *Mol. Endocrinol.* **14**, 506–517 (2000).
25. Szyf, M., Weaver, I. C. G., Champagne, F. A., Diorio, J. & Meaney, M. J. Maternal programming of steroid receptor expression and phenotype through DNA methylation in the rat. *Front. Neuroendocrinol.* **26**, 139–162 (2005).
26. Holekamp, K. ., Smale, L. & Szykman, M. Rank and reproduction in the female spotted hyaena. *J. Reprod. Fertil.* **108**, 229–237 (1996).
27. Frank, L. G., Glickman, S. E. & Powch, I. Sexual dimorphism in the spotted hyaena (Crocuta crocuta). *J. Zool.* **221**, 308–313 (1990).
28. Smith, J. E. & Holekamp, K. E. Landmark Studies: Spotted Hyenas. *Encycl. Anim. Behav.* 335–349 (2010). doi:10.1016/j.cub.2006.10.009
29. Smale, L., Frank, L. G. & Holekamp, K. E. Ontogeny of dominance in free-living spotted hyaenas: juvenile rank relations with adult females and immigrant males. *Anim. Behav.* **46**, 467–477 (1993).
30. Holekamp, K. E. & Smale, L. Ontogeny of dominance in free-living spotted hyaenas: juvenile rank relations with other immature individuals. *Anim. Behav.* **46**, 451–466 (1993).
31. Engh, A. L., Esch, K., Smale, L. & Holekamp, K. E. Mechanisms of maternal rank 'inheritance' in the spotted hyaena, Crocuta crocuta. *Anim. Behav.* **60**, 323–332 (2000).
32. Green, D. S., Johnson-Ulrich, L., Couraud, H. E. & Holekamp, K. E. Anthropogenic disturbance induces opposing population trends in spotted hyenas and African lions. *Biodivers. Conserv.* **27**, 871–889 (2018).
33. Perng, W. *et al.* Micronutrient status and global DNA methylation in school-age children. *Epigenetics* **7**, 1133–1141 (2012).
34. Boeke, C. E. *et al.* Gestational intake of methyl donors and global LINE-1 DNA methylation in maternal and cord blood: Prospective results from a folate-replete population. *Epigenetics* **7**, 253–260 (2012).
35. Bates, D., Maechler, M., Bolker, B. & Walker, S. lme4: Linear mixed-effects models using Eigen and S4. (2014).
36. Lea, A. J., Altmann, J., Alberts, S. C. & Tung, J. Resource base influences genome-wide DNA methylation levels in wild baboons (Papio cynocephalus). *Mol. Ecol.* **25**, 1681–1696 (2016).
37. Breton, C. V. *et al.* Small-magnitude effect sizes in epigenetic end points are important in children's environmental health studies: The children's environmental health and disease prevention research center's epigenetics working group. *Environ. Health Perspect.* **125**, 511–526 (2017).
38. Laubach, Z. M. *et al.* Socioeconomic status and DNA methylation from birth through mid-childhood: a prospective study in Project Viva. *Epigenomics* (2019). doi:10.2217/epi-2019-0040

39. Van Horn, R. C., Engh, A. L., Scribner, K. T., Funk, S. M. & Holekamp, K. E. Behavioural structuring of relatedness in the spotted hyena (Crocuta crocuta) suggests direct fitness benefits of clan-level cooperation. *Mol. Ecol.* **13**, 449–458 (2004).
40. Amadeu, R. R. *et al.* AGHmatrix: R package to construct relationship matrices for autotetraploid and diploid species: A blueberry example. *Plant Genome* **9**, 1–8 (2016).
41. Kent, W. J. *et al.* The Human Genome Browser at UCSC. *Genome Res.* **12**, 996–1006 (2002).
42. Kinney, S. M. *et al.* Tissue-specific distribution and dynamic changes of 5-hydroxymethylcytosine in mammalian genomes. *J. Biol. Chem.* **286**, 24685–24693 (2011).
43. Ball, M. P. *et al.* Targeted and genome-scale strategies reveal gene-body methylation signatures in human cells. *Nat. Biotechnol.* **27**, 361–368 (2009).
44. Greenberg, J. R. Developmental flexibility in spotted hyneas (Crocuta crocuta): The role of maternal and anthropogenic effects. (Michigan State University, 2017).
45. van Iterson, M. *et al.* Controlling bias and inflation in epigenome- and transcriptome-wide association studies using the empirical null distribution. *Genome Biol.* **18**, 1–13 (2017).
