# Supplementary material for: Early-life social experience affects offspring DNA methylation and later life stress phenotype
Source: Nat Commun. 2021 Jul 20;12:4398. doi: 10.1038/s41467-021-24583-x (PMC8292380; doi:10.1038/s41467-021-24583-x)
Supplement: Supplementary file 3 — Reporting Summary [file 41467_2021_24583_MOESM3_ESM.pdf]

nature research

Corresponding author(s): Zachary M. Laubach

Last updated by author(s): May 27, 2021

Reporting Summary

Nature Research wishes to improve the reproducibility of the work that we publish. This form provides structure for consistency and transparency in reporting. For further information on Nature Research policies, see our Editorial Policies and the Editorial Policy Checklist.

Statistics

For all statistical analyses, confirm that the following items are present in the figure legend, table legend, main text, or Methods section.

n/a	Confirmed	
☐	☒	The exact sample size (*n*) for each experimental group/condition, given as a discrete number and unit of measurement
☐	☒	A statement on whether measurements were taken from distinct samples or whether the same sample was measured repeatedly
☐	☒	The statistical test(s) used AND whether they are one- or two-sided<br>*Only common tests should be described solely by name; describe more complex techniques in the Methods section.*
☐	☒	A description of all covariates tested
☐	☒	A description of any assumptions or corrections, such as tests of normality and adjustment for multiple comparisons
☐	☒	A full description of the statistical parameters including central tendency (e.g. means) or other basic estimates (e.g. regression coefficient) AND variation (e.g. standard deviation) or associated estimates of uncertainty (e.g. confidence intervals)
☐	☒	For null hypothesis testing, the test statistic (e.g. *F*, *t*, *r*) with confidence intervals, effect sizes, degrees of freedom and *P* value noted<br>*Give P values as exact values whenever suitable.*
☒	☐	For Bayesian analysis, information on the choice of priors and Markov chain Monte Carlo settings
☒	☐	For hierarchical and complex designs, identification of the appropriate level for tests and full reporting of outcomes
☐	☒	Estimates of effect sizes (e.g. Cohen's *d*, Pearson's *r*), indicating how they were calculated

Our web collection on statistics for biologists contains articles on many of the points above.

Software and code

Policy information about availability of computer code

Data collection	The full data used in this paper are available at https://zenodo.org/badge/latestdoi/######## (DOI: ##) ref. Source data are provided with this paper.
Data analysis	R version 4.0.2 (2020-06-22), Platform: x86_64-apple-darwin17.0 (64-bit), Running under: macOS Catalina 10.15.7.

For manuscripts utilizing custom algorithms or software that are central to the research but not yet described in published literature, software must be made available to editors and reviewers. We strongly encourage code deposition in a community repository (e.g. GitHub). See the Nature Research guidelines for submitting code & software for further information.

Data

Policy information about availability of data

All manuscripts must include a data availability statement. This statement should provide the following information, where applicable:

- Accession codes, unique identifiers, or web links for publicly available datasets
- A list of figures that have associated raw data
- A description of any restrictions on data availability

Source code is provided with this paper at https://zenodo.org/badge/latestdoi/######## (DOI: ##) ref.

April 2020

Field-specific reporting

Please select the one below that is the best fit for your research. If you are not sure, read the appropriate sections before making your selection.

☐ Life sciences ☐ Behavioural & social sciences ☒ Ecological, evolutionary & environmental sciences

For a reference copy of the document with all sections, see nature.com/documents/nr-reporting-summary-flat.pdf

Ecological, evolutionary & environmental sciences study design

All studies must disclose on these points even when the disclosure is negative.

Study description

We used behavioral data and biological samples collected between June 1988 and July 2016 by the Mara Hyena Project, an ongoing field study of wild spotted hyenas (Crocuta Crocuta) in the Masai Mara National Reserve, Kenya (approved by MSU IACUC). For each hyena from our study population, we have information on demographic, social, and ecological conditions throughout their lifetimes. Using blood samples from immobilized hyenas, we constructed three primary datasets for analyses: global DNA methylation (n = 186 total; n = 99 females and n = 87 males), genome-wide DNA methylation (n = 29 total; n =29 females and n = 0 males), and candidate gene DNA methylation (n = 78 total; n = 43 females and n = 35 males).

Research sample

Wild spotted hyenas (Crocuta crocuta) from the Masai Mara, Kenya.

Sampling strategy

Individually identifiable animals have been continuously observed since 1988. Behavior data are collected using focal animal surveys, critical incident, and scan sampling. Fecal samples are collected from known individual any time those animals are observed defecating. Blood and other biological samples are obtained during opportunistic darting events.

Data collection

Data are collected by the project PIs (Dr. Holekamp, Dr. Smale), trained postdocs and graduate students, and trained research assistants.

Early social experiences
We derived the maternal care variables from focal animal survey (FAS) data collected during observation sessions in which: 1) mother-offspring pairs were present together for a minimum of five minutes and offspring were less than 13 months old and, 2) mothers were lactating since our intention was to focus on maternal care received early in life while offspring were dependent on nursing for sustenance. We quantified durations of maternal care behaviors from FAS data 28 based on counts of behaviors occurring during each minute of observation in which both the mother and offspring were present together. Behavioral data were collected daily between roughly 0530 – 0900 h and 1700 – 2000 h. We focused on three maternal care behaviors: minutes the mother and cub spent in close proximity (≤1 meter apart), minutes offspring spent latched to their mother's nipple (nursing), and minutes during which mothers were observed grooming (i.e., licking) their offspring. Information on additional behavioral data processing appears in the Supplementary Material.
We measured social connectivity by generating association networks among hyenas based on co-occurrences between each hyena and its group members. For each hyena, we constructed separate association networks during the CD and DI periods (two life stages during which social interactions were previously identified as key determinants of fitness29) as previously described for this population 26.
From each hyena's association networks during its CD and DI periods, we extracted three metrics that quantify how connected a hyena is with its group members: degree centrality, strength, and betweenness centrality. We focused on these metrics, rather than the overall network structure, because they reflect an individual hyena's connectedness within its network. A description of social network methods has been published in Turner et al. (2018) and is summarized in the Supplementary Material.

DNA methylation: Global (%CCGG), genome-wide, and candidate gene
We quantified global (%CCGG) DNA methylation derived from whole blood using the LUminometric Methylation Assay (LUMA) 30. Descriptions of the LUMA assay, laboratory procedures, and data cleaning protocol are available in 31, from which the current global DNA methylation samples were drawn. The majority of CpG sites assessed via LUMA occur in intergenic regions of the genome, where they may repress repetitive elements 32 and enhance chromosome stability 33, as well as introns, where they may function in transcription regulation and alternative splicing 34 (Supplementary Figure 8). Coupled with evidence that global DNA methylation is responsive to early-life environmental factors 35–37, and the fact that lower global DNA methylation is associated with a range of adverse health outcomes in general-risk human populations (e.g., shorter telomere length as a metric of accelerated aging 38,39; hypertension 40; and chronic obstructive pulmonary disease 41), we interpret average %CCGG methylation as a relevant biomarker of developmental plasticity where lower global DNA methylation is disadvantageous to health.
We measured genome-wide DNA methylation at a single nucleotide resolution in whole blood collected from hyenas 11-27 months old. To prepare the multiplexed Enhanced Reduced Representation Bisulfite Sequencing (mERRBS) sample library, we followed the protocol of Garrett-Bakelman et al. 42 using 100ng of high-quality genomic DNA. Each sample was spiked with 1ng of non-methylated Lambda gDNA to estimate bisulfite conversion efficiency. Five libraries were pooled per lane of an Illumina HiSeq4000® platform for single-end sequencing with a 50-nucleotide read length. After DNA sequencing, we ran a standard bioinformatic pipeline to clean, align, and call DNA methylation reads using the draft spotted hyena genome 43. We filtered read counts to a minimum of 10x coverage. Details on DNA library preparation and the bioinformatics pipeline are in the Supplementary Material.
Given the extensive literature on the relevance of the glucocorticoid receptor (GR) gene to both early social experiences and stress phenotypes, we also assessed CpG methylation in the putative GR promoter region of DNA from hyenas. Candidate gene bioinformatics and laboratory methods are described in the Supplementary Material. We identified CpG sites in hyena DNA that aligned with those in DNA from humans and rats (Supplementary Figures 2-6; 44,45). We calculated CpG site-specific DNA methylation in the putative hyena GR promoter region using pyrosequencing.

Fecal glucocorticoid metabolites (fGCMs)

Since January 1993, we have opportunistically collected fecal samples any time an individually identifiable hyena was seen defecating. Fecal samples were mixed and transferred to 2mL cryovials before flash freezing in liquid nitrogen within 12 h of collection. The frozen samples were then transported from our field site to the U.S. Here, we focus on the hormone corticosterone, as indicated by the concentration of fecal glucocorticoid metabolites (fGCMs) measured via a validated hormone extraction process and radioimmunoassay developed for our study population 46,47.

Demographic, social experience, and ecological covariates
We considered three categories of potential confounding variables (i.e., those that are associated with the explanatory variable but not a cause of the explanatory variable and potential determinants of the dependent variable) and included them in multiple variable models to improve causal inference. Sex was a demographic confounder; maternal rank, litter size, parity and clan size were social experience confounders; and human disturbance and local prey abundance were ecological confounders. Prey abundance was defined as either high or low, respectively, depending on whether or not the annual mass migration of wildebeest and zebra was present in the study area. The Supplementary Material provides details on data collection germane to these variables.

Statistical modeling framework
We analyzed data in four distinct parts in accordance with our goals and using a methodical approach to make causal inference from observational data 48. First, we characterized the relationship between early social experience according to maternal care and social network metrics (explanatory variables) and adult stress phenotype as indicated by adult fGCMs (dependent variable). Second, we examined associations between early social experience and global DNA methylation, and between global DNA methylation and fGCMs. Third, given that global DNA methylation may be a potential mechanism (in addition to an outcome), we conducted a formal mediation analysis following Baron & Kenny's method (1986) on a subset of hyenas (n = 30 maternal care and n = 52 social networks) for which we had data on early social experience, global DNA methylation, and adult fGCMs (Supplementary Figure 7). Fourth, to complement the third objective, we used genome-wide DNA methylation data to identify potential functional biomarkers that might link early-life experiences with future stress phenotype and that might be formally assessed as mediators in future analyses. In all models, we considered ? = 0.05 as the threshold for statistical significance, unless otherwise indicated.

Timing and spatial scale

We used behavioral data and biological samples collected between 1988 and 2016 by the Mara Hyena Project, an ongoing field study of wild spotted hyenas (Crocuta Crocuta) in the Masai Mara National Reserve, Kenya.

Data exclusions

There are four primary data sets used in this manuscript. We describe each data set hear and provide additional details on cleaning steps for each data set are described in the supplementary material. We analyzed data in four distinct parts in accordance with our goals and using a methodical approach to make causal inference from observational data. First, we characterized the relationship between early social experience according to maternal care and social network metrics (explanatory variables) and adult stress phenotype as indicated by adult fGCMs (dependent variable). Second, we examined associations between early social experience and global DNA methylation, and between global DNA methylation and fGCMs. Third, given that global DNA methylation may be a potential mechanism (in addition to an outcome), we conducted a formal mediation analysis following Baron & Kenny's method (1986) on a subset of hyenas (n = 30 maternal care and n = 52 social networks) for which we had data on early social experience, global DNA methylation, and adult fGCMs (Supplementary Figure 7). Fourth, to complement the third objective, we used genome-wide DNA methylation data to identify potential functional biomarkers that might link early-life experiences with future stress phenotype and that might be formally assessed as mediators in future analyses. In all models, we considered alpha = 0.05 as the threshold for statistical significance, unless otherwise indicated.

Our maternal care data included 1532 FAS totaling approximately 798 hours of observations of 258 mother-infant pairs when offspring were ≤1 year old, which is the approximate age at weaning in this sample (11.59 months) as well as among spotted hyenas more generally 1. We filtered these data to include only FAS sessions during which 1) the mother was lactating and 2) the mother and offspring were observed together for a minimum of five minutes. We focused on three specific maternal care behaviors: time spent in close proximity, nursing, and grooming. We chose these specific metrics of maternal care because comparable behaviors in primates and rodents have been shown to influence offspring behavior and physiology, albeit in captive settings. Additionally, these behaviors capture the effect of nutrition as well as physical contact on offspring development. Nota bene, maternal care behaviors were not mutually exclusive in their occurrence; for example, a portion of time spent in close proximity includes time spent nursing or grooming. The FAS data were collected during four distinct study periods between 1988 and 2013. Therefore, we checked for consistency between the four periods of data collection by visually inspecting a principal component analysis (PCA) plot (Supporting Figure 1). We saw no evidence of clustering by sample collection period and concluded that variation in maternal care was likely not confounded by sampling batch effects, so we were able to pool data from all study periods together.
Maternal care behaviors from focal animal survey (FAS)

Social network metrics derived from 115 hyenas during the communal den dependent (CD) and communal den independent (DI) periods of development
Association social networks were based on regular 15-20 minute interval scan sampling during observations sessions in which two or more hyenas were present 5. The networks incorporated twice weighted association index data 6, which enabled us to correct for sampling bias that could stem from variation in hyena group observability, thus providing more reliable information about social bonds in our population 5,7. In our association networks, degree centrality (i.e., degree) corresponded to the number of different individuals with which the hyena was recorded in the same session. Strength, a weighted metric of degree or network connectedness 8, corresponded to the total number of times the hyena was observed with other clan mates, including repeated associations with the same individuals. Betweenness centrality (i.e., betweenness) was calculated as the number of shortest paths that connected hyenas in the group and passed through the hyena of interest 8. An individual with high betweenness can be thought of as a bridge by which otherwise unconnected members of the network are indirectly connected. As with maternal care, we z-score standardized all association index social network metrics prior to using these measures as explanatory variables in downstream models.

Blood collection, processing, and storage
Hyenas from our study population were immobilized using a CO_2 rifle that propelled a pressurized dart containing 6.5 mg/kg of tiletamine-zolazepam (Telazol ®). After the hyena was sedated, we drew blood from their jugular vein into ethylenediaminetetraacetic acid (EDTA) coated vacuum tubes. We flash froze whole blood samples in liquid nitrogen or we extracted genomic DNA using Gentra Pure Gene or PAXgene Blood DNA kits by Qiagen® and stored samples until they were transported to the U.S. for long term storage at -80?.

We organized our analyses into four parts and included two primary data sets for both global and genome-wide DNA methylation. The global DNA methylation data set used in our analyses comprises four overlapping data subsets with information on: (1) DNA methylation measures from 186 cub and subadult hyenas (age ≤24 months), (2) maternal care behaviors from focal animal survey (FAS) data on 258 unique mother-cub pairs, (3) social network data from 115 hyenas during two early periods of development: the communal den (CD) period when a young hyena resided exclusively at the communal den, and the den independent (DI) period, which began when cubs were found away from the communal den on at least 4 consecutive occasions, and (4) fecal Glucocorticoid Metabolites (fGCMs) from 268 adult (≥24 months old) hyenas. The overlap among data sets and the final analytical samples sizes for analysis parts one through three are shown in Supplementary Figure 7. It is also worth noting that the earliest date of the maternal care FAS session and the start date of the social network period preceded the immobilization date when blood was drawn, and DNA methylation was measured. We also included only fGMCs that were obtained after assessments of maternal care behaviors, social network metrics, and %CCGG DNA methylation in order to preserve temporal relationships between our explanatory variables and our outcome variables to improve causal inference.

The fourth part of our analysis focused on our genome-wide DNA methylation data. Using mERRBS, we assayed 29 hyenas, 25 of which also had fGCMs, and which additionally had information on maternal rank (n = 23) and maternal care metrics from FAS (n = 9). Similar to the maternal care metrics, we summarized repeated measures of fGCMs via a mixed-effects linear regression model in which the natural log of fGCMs was the dependent variable. We controlled for each hyena's age in months, reproductive state, and time of day (AM vs PM) when the fecal sample was collected, a random intercept for offspring ID to account for correlations between samples collected from the same individual, and an unstructured covariance matrix. Repeated fGCMs were limited to when hyenas were 1 yr or older so that our assessment of the stress phenotype roughly overlapped or proceeded the assessment of genome-wide DNA methylation. We then calculated BLUPS from this mixed-effects model. The BLUPs effectively represent each hyena's deviation in fGCMs relative to the population average after accounting for key demographic covariates. This method has been previously used to consolidate repeated measurements of a given variable into a single value per individual without making assumptions about the underlying distribution of the data 33,34. Linear mixed-models were run using the R package lme4 35.
Prior to conducting our epigenome wide association study (EWAS), we filtered the mERRBS data set. In order to reduce the type I error rate and the burden of multiple comparisons correction, we removed 5% of CpG sites that had the lowest inter-individual variation of DNA methylation 36. We also excluded CpG sites with low (<10%) and high (>90%) average DNA methylation. While this filtering should increase the signal to noise ratio in our analysis, there is a trade off in which some true but small effect size associations may not be captured. We note this limitation given that we and others have reported and discussed the biological relevance of small differences in DNA methylation, particularly in developmental origins studies 37,38.
We accounted for relatedness among individuals comprising our study population by including a genetic covariance matrix in our EWAS models. We built the genetic covariance matrix by combining our extensive hyena lineage data 7 with paternity data determined from an established method utilizing microsatellites from DNA extracted from blood and fecal samples 39. We determined paternity for 24 of 29 individuals and maternal ID was known for all study animals. With these data we constructed a pedigree and used the R package, 'AGHmatrix,' 40 to calculate a relatedness A-matrix, which we used to control for genetic covariance in our EWAS.

Reproducibility	NA as this was an observational study
Randomization	This was an observational study that had 4 main analytical parts. Extensive details are provide in the manuscript, including directed acyclic graphs, that explicitly map out which variables were controlled for in each model.
Blinding	Data were collected prospectively and not of the exclusive purpose of this study. Therefore those collecting data were blind to the study design and hypotheses tested here.

Did the study involve field work? ☒ Yes ☐ No

Field work, collection and transport

Field conditions	Field conditions vary given that these data were collected over more than 20 years. In general, the Mara is a savannah ecoystem, located near the equator, and that experiences biannual wet and dry seasons.
Location	Masai Mara, Kenya, is located at approximately, latitude -1.490000 and longitude 35.143890 and an elevation of ~1600m above sea level.
Access & import/export	All sample exports are in accord with agreements between Michigan State University, the US Fish and Wildlife Serivce, Kenya Wildlife Service, and additional regional and local government authorities in Kenya.
Disturbance	Most of our data collection is non-invasive. We minimze discomfort of animals that are darted in by following our extensive darting protocol that has been approved by MSU IACUC and Kenyan Wildlife Service veterinarians.

Reporting for specific materials, systems and methods

We require information from authors about some types of materials, experimental systems and methods used in many studies. Here, indicate whether each material, system or method listed is relevant to your study. If you are not sure if a list item applies to your research, read the appropriate section before selecting a response.

Materials & experimental systems

n/a	Involved in the study
☒	☐ Antibodies
☒	☐ Eukaryotic cell lines
☒	☐ Palaeontology and archaeology
☐	☒ Animals and other organisms
☒	☐ Human research participants
☒	☐ Clinical data
☒	☐ Dual use research of concern

Methods

n/a	Involved in the study
☒	☐ ChIP-seq
☒	☐ Flow cytometry
☒	☐ MRI-based neuroimaging

Animals and other organisms

Policy information about studies involving animals; ARRIVE guidelines recommended for reporting animal research

Laboratory animals	No laboratory animals were used in the study
Wild animals	Spotted hyenas (Crocuta crocuta) were observed in their natural habitat. The study population include mixed age (cub-adult) and mixed sex animals.
Field-collected samples	We used data from both male and female wild spotted hyenas (Crocuta crocuta) from the Masai Mara, Kenya. We collected non-invasive fecal samples and behavioral data from our study animals. As part of our long-term data collection, we routinely darted study animals in order to collect biological samples and morphological measurements. Of special relevance to this study is our blood collection procedure. We immobilized hyenas using 6.5 mg/kg of tiletamine-zolazepam (Telazol ®) in a pressurized dart fired from a CO2 powered rifle. We then drew blood from the jugular vein into sodium heparin-coated vacuum tubes. After the hyena was secured in a safe place to recover from the anesthesia, we took the samples back to camp where a portion of the collected blood was spun in a centrifuge at 3000 rpm for 10 minutes to separate red and white blood cells from plasma. Plasma was aliquoted into multiple cryogenic vials. Immediately, the blood derivatives, including plasma, were flash frozen in liquid nitrogen where they remained until they were transported on dry ice to a -80°C freezer in the U.S. All samples remained frozen until time of laboratory analysis for the T. gondii assays.opportunistically dart our study animals as previously described.
Ethics oversight	This study was approved by MSU IACUC

Note that full information on the approval of the study protocol must also be provided in the manuscript.
